# Denaturing mass photometry for rapid optimization of chemical protein-protein cross-linking reactions

**DOI:** 10.1038/s41467-024-47732-4

**Published:** 2024-04-25

**Authors:** Hugo Gizardin-Fredon, Paulo E. Santo, Marie-Eve Chagot, Bruno Charpentier, Tiago M. Bandeiras, Xavier Manival, Oscar Hernandez-Alba, Sarah Cianférani

**Affiliations:** 1 Laboratoire de Spectrométrie de Masse BioOrganique, IPHC UMR 7178, Université de Strasbourg, CNRS, Strasbourg, France; 2 Infrastructure Nationale de Protéomique ProFI – FR2048, Strasbourg, France; 3 iBET, Instituto de Biologia Experimental e Tecnológica, Apartado 12, 2781-901, Oeiras, Portugal; 4 Instituto de Tecnologia Química e Biológica António Xavier, Universidade Nova de Lisboa, Av. da República, 2780-157, Oeiras, Portugal; 5 IMoPA, CNRS, Université de Lorraine, Nancy, France

**Keywords:** Protein-protein interaction networks, Mass spectrometry, Single-molecule biophysics

## Abstract

Chemical cross-linking reactions (XL) are an important strategy for studying protein-protein interactions (PPIs), including low abundant sub-complexes, in structural biology. However, choosing XL reagents and conditions is laborious and mostly limited to analysis of protein assemblies that can be resolved using SDS-PAGE. To overcome these limitations, we develop here a denaturing mass photometry (dMP) method for fast, reliable and user-friendly optimization and monitoring of chemical XL reactions. The dMP is a robust 2-step protocol that ensures 95% of irreversible denaturation within only 5 min. We show that dMP provides accurate mass identification across a broad mass range (30 kDa–5 MDa) along with direct label-free relative quantification of all coexisting XL species (sub-complexes and aggregates). We compare dMP with SDS-PAGE and observe that, unlike the benchmark, dMP is time-efficient (3 min/triplicate), requires significantly less material (20–100×) and affords single molecule sensitivity. To illustrate its utility for routine structural biology applications, we show that dMP affords screening of 20 XL conditions in 1 h, accurately identifying and quantifying all coexisting species. Taken together, we anticipate that dMP will have an impact on ability to structurally characterize more PPIs and macromolecular assemblies, expected final complexes but also sub-complexes that form en route.

## Abstract (web summary)

Choosing best chemical cross-linking (XL) reagents and conditions for studying protein-protein interactions in structural biology is laborious and lacks in accuracy. The authors develop here an accurate, fast, robust and quantiative denaturing mass photometry approach for screening of XL conditions.

## Introduction

Vast majority of biological processes that drive life depend on formation of specific protein-protein interactions (PPIs). Thus, characterizing PPIs has been one of the cornerstones of structural biology for decades, and many structures of macromolecular assemblies are now available and allow detailed analysis of factors that determine interactions between macromolecules. However, many physiologically relevant PPIs form only transiently, and these have been notoriously difficult to capture and subject to structural analysis. Protein cross-linking (XL) uses chemical reagents to introduce covalent bonds between residues from two or more proteins that are in close proximity, i.e. involved in a PPI interface, and is a routinely used strategy to overcome this challenge^1–6^. For example, XL is frequently used before electron microscopy (EM) to stabilize complexes or targeted conformations, and in mass spectrometry workflows (XL-MS) in order to identify regions of proteins that are in close proximity. In general, the first step of the chemical XL protocol is of utmost importance as its success drives the outcome of the biophysical measurement^7^. Therefore, the use of XL requires careful optimization of XL conditions to ensure that PPIs are accurately captured and preserved throughout subsequent structural analysis, and that generation of “biologically non-specific” XL aggregates is minimal^8^. The optimization steps routinely include selection of the XL reagent, among dozens available, and screening XL reaction conditions that are often dependent on the specific PPI under investigation, resulting in time-consuming and fastidious nature of these steps. For both XL-MS^9^ and EM sample preparation, denaturing sodium dodecyl sulfate–polyacrylamide gel electrophoresis (SDS-PAGE) is recommended to monitor and optimize XL conditions, as it allows visualization of high molecular weight (MW) cross-linked species and concomitant vanishing of the bands corresponding to individual protein partners. Although SDS-PAGE is widely available, easy to use, and affordable, this method: i) allows only rough estimate of MWs; ii) is not suitable for analysis of high MW complexes as they don’t enter the gel; iii) is time consuming (gel casting, sample denaturation, gel migration, staining); and iv) is intrinsically low throughput. Moreover, given these limitations of SDS-PAGE, outcomes of XL reactions are often obtained only after additional biophysical evaluation, further complicating the process. Therefore, more accurate and rapid methods to identify the covalently-stabilized species generated upon chemical XL represent a major need for the field.

Mass photometry (MP) is an emerging single-molecule biophysical technique^10^ that operates under native conditions (nMP) to allow analysis of protein complexes recalcitrant to native mass spectrometry (nMS), *i.e*. “nMS-resistant” protein complexes^10–13^. MP is based on the principle of the interferometric scattering microscopy (iSCAT) that uses the contrast generated as a result of the destructive interference between the scattered light and reflected light of biomolecules in solution upon irradiation with a visible laser light^14–16^. As the contrast intensity linearly scales with the mass, MP can serve to estimate masses of biomolecules after proper calibration with reference molecules. Importantly, MP analysis requires simple sample preparation, takes only minutes to complete using small amounts of sample (100 pM–100 nM) without prior buffer exchange, displays broad mass range (30 kDa to 5 MDa), and yet allows multiplexing and automation^11,17^. Additionally, single molecule detection enables relative quantification of detected populations, which can be used to estimate affinity constants in the nM-µM concentration range^18,19^. Although nMS mass accuracy remains superior, nMP has emerged as a valuable asset for characterizing highly heterogeneous complexes^20^, membrane proteins solubilized with different types of membrane mimics^11,21,22^, ribosomes^12^, and viral capsids^13,23^. In addition to analyzing native-like complexes, nMP was recently used in combination with chemical cross-linking (XL) to verify stabilization of an XL-ed oligomer before EM experiments^20^. These advantages of nMP and compatibility with chemical XL conditions, suggest that developing a denaturing MP (dMP) technique may offer opportunities for improved and rapid optimization of chemical XL reaction conditions, and overcome current limitations of SDS-PAGE.

In this work, we develop an accurate and robust single-molecule protocol to perform MP analysis in denaturing conditions. We first use reference protein complexes of increasing sizes and complexities as proof-of-concept, and for dMP performance assessment. We then evaluate our dMP protocol for XL reaction monitoring and benchmark it against the reference gold standard denaturing SDS-PAGE gel method. Due to its single molecule detection capabilities, dMP is more precise than conventional SDS-PAGE analysis, providing accurate mass identification as well as relative quantification of all coexisting XL-ed species. Thus, dMP represents an improved technique to monitor and optimize XL reactions through large screens, as we illustrate here using XL-MS study of R2SP complex interactions.

## Results

### Development of a denaturing mass photometry (dMP) protocol

**Fig. 1:**
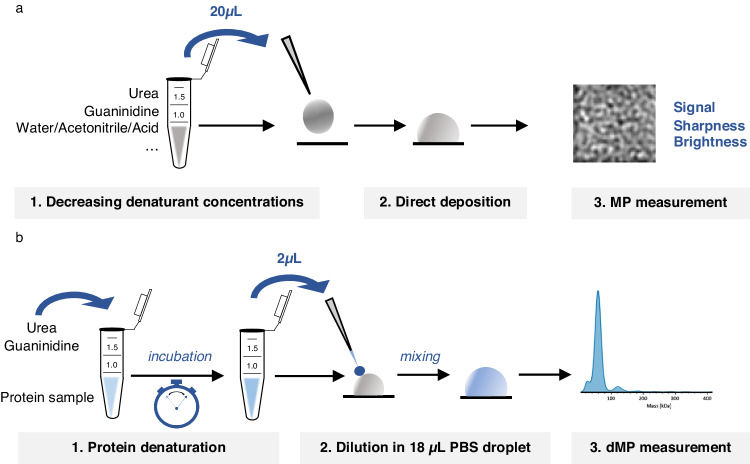
Schemes representing two MP-based protocols used during dMP method development. **a** Scheme of the assay for the evaluation of denaturing agents’ compatibility with MP measurements, **b** Optimized general workflow for dMP analysis.

To develop a fast, efficient and non-reversible denaturation protocol while maintaining good quality MP signal intensities, we optimized several sample preparation parameters. First, we focused on the choice of the denaturing agent and examined effects of urea and guanidine hydrocloride (GdnHCl), two well-known and widely used protein denaturants^24–26^, as well as H_2_O/ACN/FA mix (50/50/1), which is classically used in intact MS mass measurement under denaturing conditions. We observed that H_2_O/ACN/FA was not compatible with stabilization of MP droplets, and was not pursued further. We next assessed the impact of urea/GdnHCl solutions on the quality of MP signal using “protein-free” droplets (Fig. 1a) by monitoring three output indicators (signal-*Si*, sharpness-*Sh* and brightness-*Br*) that reflect the quality of the MP images/frames. In general, *Si* reports on the level of activity in each frame, which can be due to either protein binding, or contaminants/salts/surfactants presence, and its values should be as low as possible (<0.05%) to avoid extensive peak broadening^17^. *Sh* refers to the level of detail visible in each frame, which impacts the ability to find and maintain the good focusing position, and its value should be as high as possible. Finally, *Br* characterizes the amount of light available in images, and its value should be maximized to avoid peak broadening. With the aim to reach similar MP signal quality as the one reached in Phosphate Buffer Saline (PBS) droplets (*Si* ∼0.03%, *Sh* ∼5%, *Br* ∼73%), we used the “buffer-free” focusing mode to directly analyze protein-free droplets containing decreasing concentrations of urea or GdnHCl (from 5.4 to 0.4 M). Independently of the denaturing agent, *Sh* increased progressively from 1 to 3% at 5.4 M of urea/GdnHCl to ≥ 5% at lower concentrations (Supplementary Table 1). Similarly, *Br* increased at lower denaturing agent concentrations with an optimal value of ∼67% obtained at 0.8 M urea/0.4 M GdnHCl. *Si* values were <0.05% for all tested concentrations lower than 5.4 M. Altogether, our “protein-free” blank MP acquisitions allowed establishing the optimal concentrations of denaturing agents (<0.8 M of urea or GdnHCl). As XL reactions are typically conducted in the nM-µM protein concentration range, these samples need to be diluted (approximatively 10× dilution in PBS) prior to dMP measurements. That means that the initial concentration of urea or GdnHCl during the denaturing reaction can be set at 5.4 M and 6 M, respectively, without exceeding the 0.8 M concentration limit in the final droplet.

To further optimize our dMP protocol, we used reference protein complexes (BSA, ADH, GLDH, 20 S proteasome) to either assess mass precision, accuracy and peak broadening in dMP (BSA), or to further optimize the denaturation step (ADH, GLDH and 20 S proteasome). After Gaussian-fitting of MP histograms, the mean mass (µ) and half-height peak width (2σ, FWHM*)* of the Gaussian fits were used to evaluate mass accuracy and peak broadening, respectively. Considering dMP triplicate measurements, the measured mass of BSA oligomers denatured in urea and GdnHCl was the same as the one obtained using nMP measurements (Supplementary Table 2). In addition, our denaturation protocol does not alter MP variability between replicates, with SDs ≤ 3 kDa and ≤5 kDa for BSA monomers and dimers, respectively. Finally, denaturation only slightly affected peak broadening (FWHM between 8–10 kDa and 8–14 kDa, compared to 8 kDa in nMP for monomers and dimers, respectively), demonstrating that dMP performance was comparable to nMP.

**Fig. 2:**
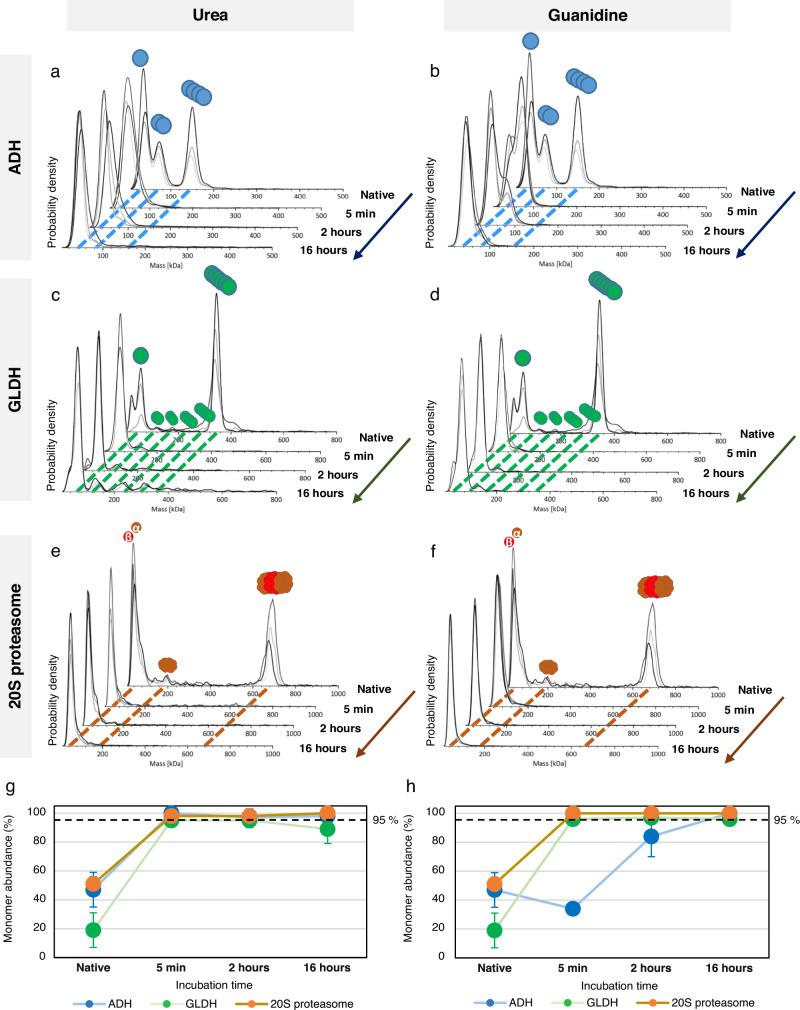
Optimization of denaturation step for dMP using ADH, GLDH and 20 S proteasome. Mass distributions, represented as probability density, show the evolution of monomer abundances, with measurement replicates (*n* = 3) shown as overlapping curves in shades of gray. MP profiles have been measured after 5 min, 2 h, and 16 h denaturation for: ADH **a** in 5.4 M urea or **b** in 6 M guanidine HCl; GLDH **c** in 5.4 M urea or **d** in 6 M guanidine HCl and 20S proteasome **e** in 5.4 M urea or **f** in 6 M guanidine HCl. Scatter plots represent the monomer abundance (mean ± SD) after ADH, GLDH and 20S denaturation with **g** urea and **h** guanidine. Standard deviations come from measurements replicate (*n* = 3). Source data are provided as a Source Data file.

In order to develop a fast denaturing protocol, we next optimized the duration of the denaturation step on proteins of increasing sizes and complexities (ADH, GLDH and 20S proteasome). Incubation in urea and GdnHCl were carried out for 5 min to 16 h at room temperature (Fig. 2a–f). After 5 min of urea denaturation, an almost complete denaturation is observed in dMP for all systems with ≥95% (Fig. 2g) of the detected species being monomers (compared to 46%, 19%, and 51% of monomers for ADH, GLDH and 20S proteasome in nMP, respectively). Conversely, denaturation in GdnHCl proved to be less efficient after 5 min for ADH (∼34% monomers) while being equivalent for GLDH and 20S proteasome (Fig. 2h), suggesting urea as a more efficient denaturing agent. Lastly, in order to ensure that no protein refolding occurs in the PBS droplet prepared for dMP measurements, and in the timeframe of the analysis, we mimicked our optimized denaturing protocol in a PBS tube (Supplementary Fig. 1a). As expected, only GLDH monomer (61 ± 6 kDa) is detected even after 10 min dilution in PBS, with perfectly superimposable dMP profiles (Supplementary Fig. 1b), demonstrating that no significant protein refolding will occur within the timeframe of dMP analysis (typically 1 min).

To conclude, we have developed and optimized a fast (5 min), efficient ( >95% denaturation) and non-reversible (in the timeframe of dMP measurements) denaturation protocol compatible with MP analysis, which will be further referred to as “denaturing MP protocol” (dMP). This workflow consists of a first step of denaturation (5 min in 5.4 M urea) followed by 10x dilution of the denatured sample right before dMP analysis (Fig. 1b). Obtained dMP measurements are of comparable quality with respect to mass accuracy and peak width, as those obtained in classical nMP analysis.

### dMP outperforms SDS-PAGE gel analysis for XL reaction monitoring

**Fig. 3:**
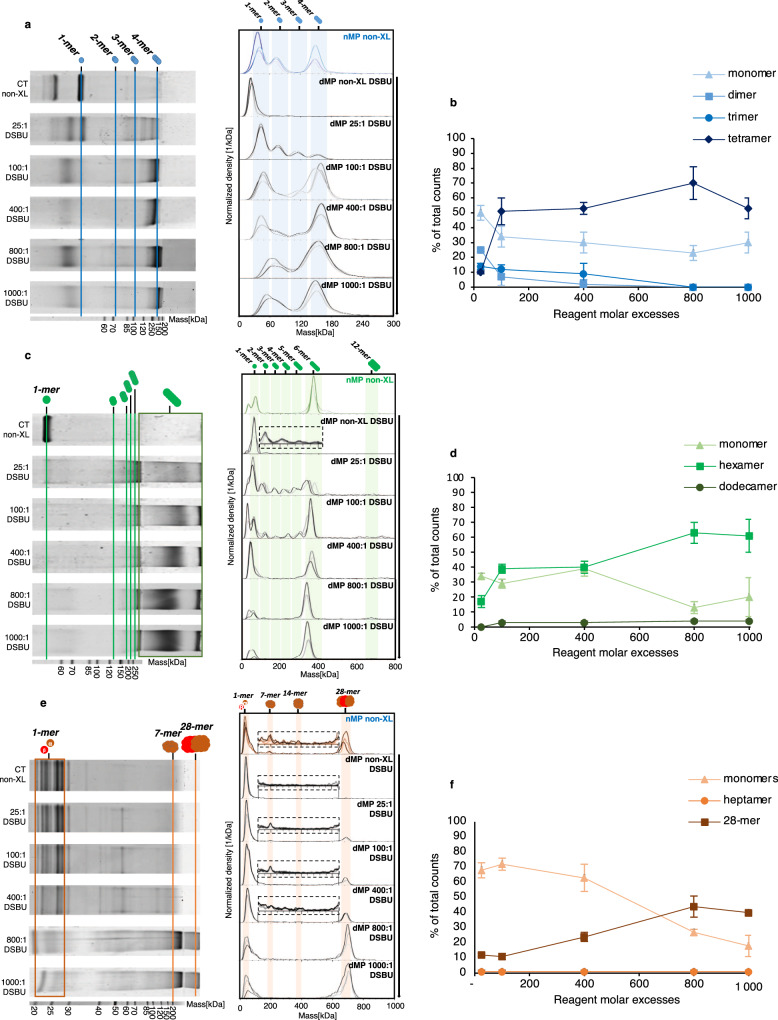
Benchmarking of dMP vs SDS-Page for the DSBU cross-linking optimization. Comparison of dMP and SDS-PAGE experiments after **a** ADH, **c** GLDH, **e** 20S proteasome cross-linking. Mass distributions are represented as probability density with overlapping curves in lighter shades, showing the measurement replicates (*n* = 3). Relative abundances of different oligomeric states of the corresponding complex are shown as scatter plots (mean ± SD) for **b** ADH, **d** GLDH, **f** 20S proteasome, with error bars representing the standard deviation related to measurement replicates (*n* = 3). Source data are provided as a Source Data file.

We next benchmarked our dMP protocol against SDS-PAGE, the gold standard for XL reaction optimization, using our reference systems: ADH tetramer (∼145 kDa), GLDH hexamer (∼313 kDa), and 20S proteasome 28-mer (∼700 kDa, see Supplementary Fig. 2 for nMP). We used disuccinimidyl dibutyric urea (DSBU; linker size ∼12.5 Å), one of the most used MS-cleavable cross-linker in XL-MS workflows as proof of concept. We tested several XL:protein concentration ratios (25:1, 100:1, 400:1, 800:1 and 1000:1), as is commonly done during XL reaction optimization. Comparing SDS-PAGE and dMP results for ADH side by side (Fig. 3a), showed that SDS-PAGE provides only a rough visualization of the products and yields of the cross-linking reaction. In contrast, dMP was able to detect monomers, dimers, trimers and tetramers at all concentration ratios, as well as allow quantification based on the relative abundance of each species. We observed that the relative yields of different assemblies (dimer vs. trimer vs. tetramer) varies with different amount of XL reagent used, as expected (Fig. 3a, b, Supplementary Fig. 3). The tetrameric species formation maxed out at about 70 ± 11% of total counts at 800 molar excesses, although even at 100:1 DSBU:ADH yields of tetramer approached this limit. No non-specific high-mass aggregates were detected either using SDS-PAGE or dMP, and both methods suggested that optimal conditions were around 100:1 DSBU:ADH.

For GLDH, the difference in performance between SDS-PAGE and dMP was even more pronounced as only one broad band in the loading-well was observed on the SDS-PAGE gel at high masses regardless of the amount of DSBU used (Fig. 3c, d). This highlights the key disadvantage of SDS-PAGE as high-mass oligomers of GLDH do not enter the gel, and as a consequence, neither clear identification nor quantification of GLDH degree of oligomerisation is possible from SDS-PAGE results. On the other hand, dMP clearly resolved all GLDH coexisting oligomeric forms and allowed quantitative monitoring of the progressive stabilization of higher oligomeric states as a function of increasing XL concentrations. We observed that, as expected, as hexamer abundance increased from at 17 ± 5% to 61 ± 11%, the concentration of intermediary sub-complexes (combination of 2-mer, 3-mer, 4-mer, 5-mer) decreased from 35 ± 5% to 7 ± 1% from low (25×) to high (1000×) DSBU molar excesses, with the small amount of dimer of hexamer (12-mer) forming at a 1000 DSBU:GLDH ratio (4 ± 1%). Lastly, the superior performance of dMP was even more obvious for cross-linked 20S proteasome (28 subunits), as SDS-PAGE was not able to detect the final product, unlike dMP that showed clear formation of the 20 S proteasome (Fig. 3e, f). Thus, dMP unambiguously detected the ∼700 kDa covalently stabilized intact 20S proteasome under all XL conditions (12 ± 1% of total abundance at 25:1 DSBU:20 S ratio, to 44 ± 7% at 800:1), as well as very low abundance ∼170 kDa 7-mer (~1% abundance in all conditions). Taken together, these results indicate that dMP outperforms SDS-PAGE in terms of mass accuracy, resolution and broader mass range (30 kDa–5 MDa for our MP instrument). Furthermore, dMP allowed accurate relative quantification of all coexisting species.

### dMP for quantitative evaluation of XL reaction performances

We next evaluated the versatility of dMP to screen for optimal chemical XL reaction using a variety XL reagents used in XL-MS workflows or for EM grid preparations. To enable quantitative assessment of the method, we defined two performance parameters: (i) total inter-protein XL reaction efficiency Eff_XL_ (Material and method Eq. (1), as an indicator of the total amount of inter-protein cross-linking that occurred (all cross-linked species beyond monomers); and (ii) “specific” XL factor SF_XL_ (Materials and Methods Eq. (2), as an estimate of the amount of the “specific” (*i.e*. expected main product) XL complex”. Eff_XL_ is thus an indicator for overall yield of the XL reaction and accounts for both specific and non-specific XL species, whereas SF_XL_ focuses only on one specific species that is the desired product (when already known). In general terms, Eff_XL_ would be a preferred metric for analysis of samples where the exact stoichiometry of the final complex might be unknown, while SF_XL_ would be more appropriate for complexes with known stoichiometries.

**Fig. 4:**
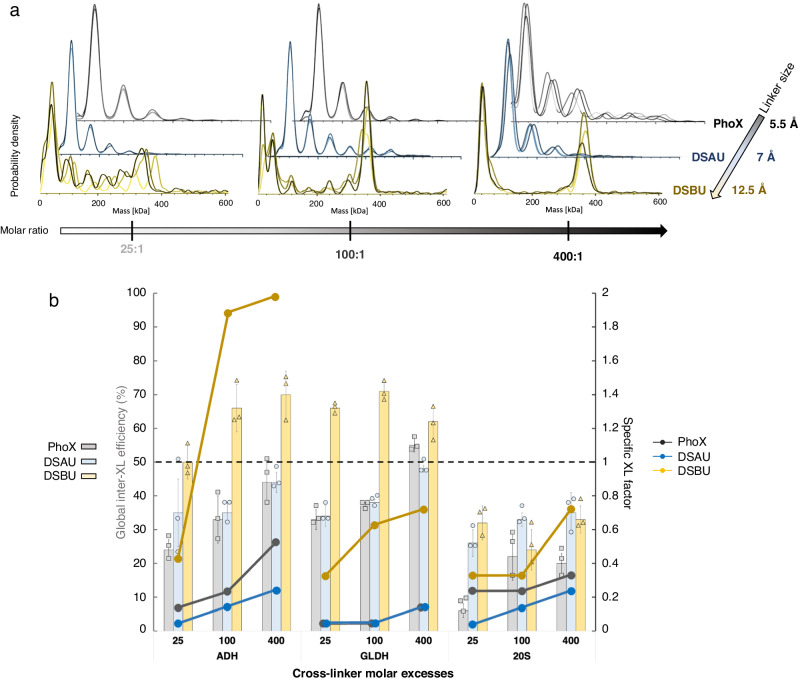
dMP screening of cross-linking conditions on ADH, GLDH and 20 proteasome. **a** Effect of cross-linking reagent (size, flexibility) on oligomeric states stabilized, measured in dMP: presented results are probability densities (KD) of GLDH samples cross-linked with increasing molar ratios of PhoX, DSAU, DSBU, from measurement replicates (*n* = 3). **b** dMP-based quantitative results of XL condition screening for ADH, GLDH, and 20S proteasome complexes. Bar charts represent the dMP-calculated global inter-XL efficiency (mean ± SD) for each complex and XL condition (25/100/400:1 cross-linker:complex molar ratio), from measurement replicates (*n* = 3). Plain dots represent the complex stabilization factor for each complex and XL condition. The black dash line corresponds to the stabilization factor value of 1 indicating a complex abundance similar to the native sample. Source data are provided as a Source Data file.

We first evaluated our dMP method for NHS-ester chemical reagents classically used in XL-MS experiments: DSBU, DSAU (disuccinimidyl diacetic urea, linker size ∼7.7 Å), and PhoX (linker size ∼5.5 Å; less-flexible IMAC-enrichable cross-linker that gained popularity for both in vitro and in vivo XL-MS studies^1,27^). For GLDH, dMP mass distributions were similar between PhoX and DSAU, both of which failed to yield hexamers even at higher concentrations (Fig. 4a). Conversely, GLDH hexamers and monomers are detected as main components at 25:1 DSBU:GLDH ratio, and this trend is even more obvious at 100:1 and 400:1 XL:GLDH ratios (Fig. 4a). Similar behaviors were also observed in dMP profiles of cross-linked ADH and 20S proteasome, with a significantly higher yield of expected oligomers with DSBU (Supplementary Fig. 4). For homo-oligomeric protein complexes (ADH tetramer and GLDH hexamer), DSBU showed much higher Eff_XL_ values (60–70%) compared to DSAU (45–48%) and PhoX (45–55%) (Fig. 4b, bar charts), suggesting overall better XL efficiencies for DSBU. For the large hetero-multiprotein 20S proteasome, trends were different as the Eff_XL_ decreased (max. ∼35%), and none of the XL reagents performed as well. In all examples studied, SF_XL_ values increased as a function of XL:complex ratio, with DSBU outperforming PhoX and DSAU (Fig. 4b, solid dots). Low SF_XL_ values combined with good Eff_XL_ (26–48%) translate DSAU abilities to generate more sub-complexes and lower amounts of expected XL-stabilized ADH, GLDH, 20S proteasome tetramers, hexamers, 28-mer. Off note, it appears that SF_XL_ values obtained for ADH and GLDH tend to plateau with increasing DSBU molar excess, which is not the case for the 20S proteasome, composed of a higher number of subunits (28) compared to AHD (4) and GLDH (6). Our dMP results on different biological systems highlight that DSBU efficiency to stabilize expected complexes/oligomers is significantly higher (SF_XL_ values close to 1) than PhoX and DSAU. These increased efficiencies go along a low abundance of sub-complexes and potential non-specific higher-masses stabilization. Importantly, the screening of the 9 different XL conditions was achieved within 30 min using only 1.8 µg of proteins.

Given that SDS-PAGE is also the gold standard for monitoring XL reaction before EM and cryoEM applications^28–30^, we tested a common XL reagent used for cryo-EM purposes, formaldehyde (FA, Supplementary Fig. 5). We carried out XL experiments of GLDH and 20S proteasome with increasing concentrations of FA (0.05% to 1%, ∼20–360 mM). Our results showed that FA starts to cross-link GLDH into hexamers at 0.2% (16 ± 2% of total counts) and 20S proteasome 28-mer at 0.05% (12 ± 1% of total counts). FA maximally cross-links GLDH and 20S proteasome at 1% concentration with 25 ± 2% and 40 ± 9% of total counts, respectively. The experiment took ∼25 min to complete per complex, therefore highlighting how dMP can facilitate rapid assessment of XL reaction yields prior to cryo-EM analysis. Taken together, these studies illustrate the power of dMP to evaluate performance of different XL reagents and reaction conditions using small amounts of sample, rapidly (in 40 min or less) and accurately (with mass resolution sufficient to distinguish between a range of different XL species), which represents a major improvement over the performance of SDS-PAGE.

### Application of dMP in integrative structural biology: the R2SP case study

**Fig. 5:**
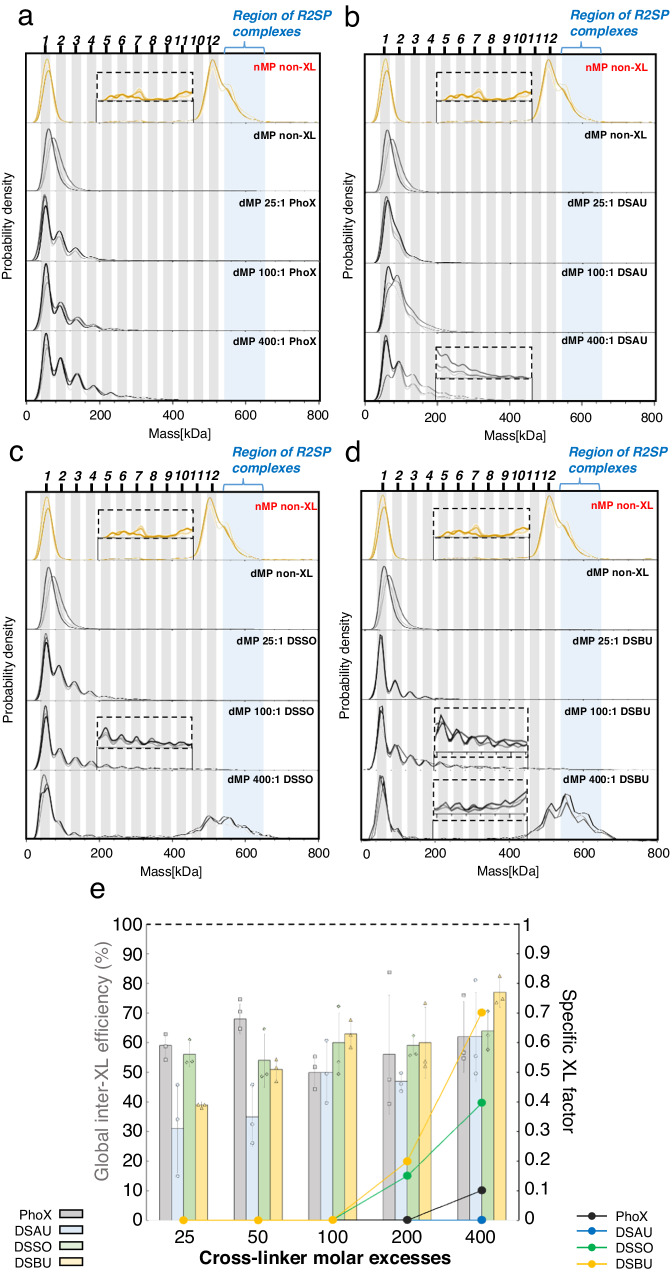
dMP results of XL reaction optimization for R2SP complex. nMP profiles (yellow) and dMP profiles (gray) of R2SP cross-linked with 0, 25, 100, 400 molar excesses of **a** PhoX, **b** DSAU, **c** DSSO, **d** DSBU, shown as probability densities. Measurement replicates (n = 3) are shown as overlapping curves in shades of yellow or gray. **e** Quantitative results of R2SP XL optimization at 25, 50, 100, 200, 400 molar excesses of reagents. Bar charts represent the dMP-calculated total inter-XL efficiency (mean ± SD) for each complex and XL condition, from measurement replicates (n = 3). Plain dots represent the specific factor for each complex and XL condition. The black dash line corresponds to the stabilization factor value of 1 indicating a complex abundance similar to the native sample. Source data are provided as a Source Data file.

To examine utility of dMP in a real-life scenario, we applied dMP to optimize XL conditions before MS analysis of R2SP, a multicomponent protein complex that includes two hexamers of 3 AAA+ ATPases RuvB-Like1 (R1) and 3 RuvB-Like2 (R2)^31^ each, 1 SPAG1 (S) molecule, and 1 PIH1D2 (P) molecule^32,33^. The first nMP measurement of the R2SP preparation allowed us to identify R1R2 hexamers at 513 ± 1.1 kDa (42 ± 10% of total counts, over triplicates, Supplementary Fig. 6) coexisting with R2SP complexes at 549 ± 4 kDa (28 ± 10% of total counts, over triplicates – expected mass ∼540 kDa, Fig. 5). We next used dMP to screen XL reaction conditions for further XL-MS analysis. We screened four different cross-linkers, PhoX, DSAU, DSSO (disuccinimidylsulfoxyde, 10.3 Å linker length) and DSBU at 5 different molar excesses (25/50/100/200/400) plus the control non-XL sample (see Supplementary Fig. 7 for complete dMP profiles). The complete analysis of 20-conditions was completed within 1 hour using dMP (including triplicate measurements), in comparison to SDS-PAGE which required 20 hours using in-house made SDS-PAGE (including gel casting, sample preparation, migration, fixation, staining, and unstaining, Supplementary Fig. 8). Furthremore, dMP used lower amounts of biological materials than SDS-PAGE (∼3 µg in total compared to ∼24 µg, respectively). Importantly, mass precision of dMP allowed us to completely resolve all the cross-linked oligomeric populations, including the low abundant ones, which allowed us to map finer differences between different XL reagents. Thus, longer/more flexible reagents (DSSO and DSBU) performed better than PhoX and DSAU, resulting in high yields of ~540 kDa R2SP complex ( > 100 molar excess, Fig. 5a, b), with Eff_XL_ ∼55–63%, max. SF_XL_ ∼0.4 for DSSO and Eff_XL_ ∼40–77%, max. SF_XL_ ∼0.7 for DSBU (see Fig. 5e), with no significant over-XL species formation. In contrast, PhoX and DSAU did not yield to R2SP stabilization (Fig. 5c, d), although they did form sub-complexes with increasing concentrations of the XL reagent (Eff_XL_ ∼50–60%, max. SF_XL_ ∼0.1 for PhoX, and Eff_XL_ ∼30–60%, SF_XL_ = 0 for DSAU, Fig. 5e). Of note, performing a nMP measurement after XL reaction confirmed that no significant denaturation resulted from the XL reaction (Supplementary Fig. 9). To conclude, dMP allowed unmatched rapidity for XL reaction condition screening in the real-life scenario using R2SP assembly as an example.

**Fig. 6:**
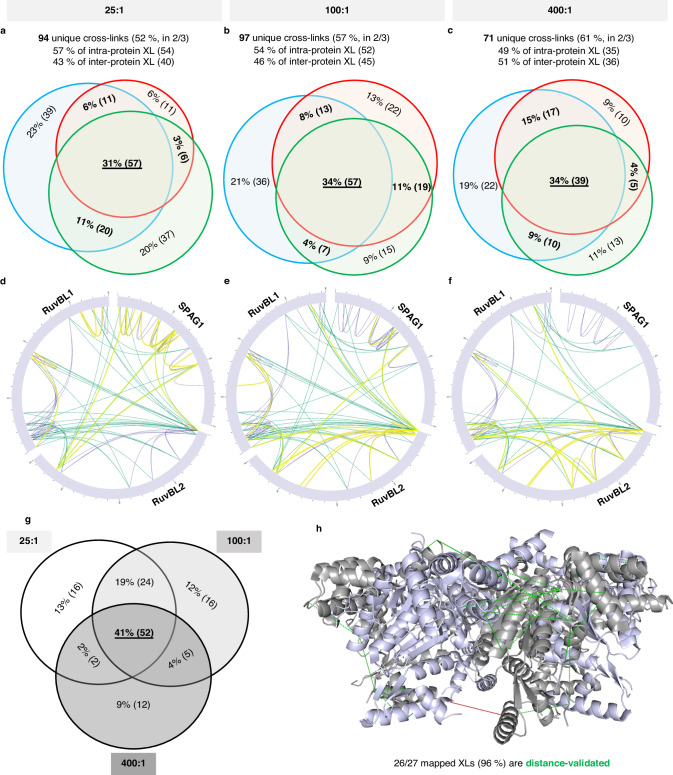
Description of the XL-MS dataset of R2SP cross-linking in triplicates with 25, 100, 400 molar excesses of DSBU. Proportional Venn diagrams show the reproducibility of cross-linking reaction across biological replicates (n = 3), at **a** 25, **b** 100, **c** 400 molar excesses of DSBU. The identified cross-links are represented in circular views^42^ along RuvBL1, RuvBL2 and SPAG1 sequences at **d** 25, **e** 100, **f** 400 molar excesses of DSBU. In yellow are highlighted the XLs unique to the condition. Venn diagram **g** shows the overlap of validated XLs (file threshold 2/3) between the 3 datasets. **h** we finally mapped identified XLs in dashed lines, on the R2SP structure (2XSZ.pdb), RuvBL1 is shown in light gray, RuvBL2 is shown in light purple.

To pursue characterization of R2SP structure further we used XL-MS, using DSBU as the XL reagent based on the results of our screening experiments. Currently, no structures of R2SP complex exist, primarily due to the difficulties in stabilizing this assembly. Thus, we conducted XL-MS experiments in triplicate on R2SP complex at 25, 100, 400 molar excesses of DSBU. In general, the XL-MS experiments capture all cross-linked peptides, both those resulting from intra- and inter-subunit cross-linking reactions. As shown in Fig. 6, we identified similar numbers of “unique XL” (defined as those XLs present in at least 2 out of the 3 replicates) at 25:1 (94) and 100:1 (97) molar excess ratios. The number of unique XLs identified at 400:1 molar excess ratio decreased to 71, which might be explained by decreased digestion efficiencies due to lysine sites congestion and difficulties for softwares to identify those highly modified peptides^34^. Nevertheless, the reproducibility (measured as the number of unique XL peptides detected in at least 2 out of 3 replicates) within all three datasets was acceptable and varied from 52% at 25:1 to 61% for 400:1 DSBU:R2P molar ratio. In terms of prevalence of intra- vs. inter-XLs, we observed that the relative% of unique inter-XLs identified increased with increasing molar ratio of DSBU, from 43% at 25:1 to 51% at 400:1 (Fig. 6a–c). Next, we mapped the identity of validated unique XLs identified using different XL conditions onto different components of the R2SP structure (Figs. 6d–g). Overall, the overlap of validated unique XLs between the three conditions represents 52 XL peptides (41% of total unique XLs, Fig. 6g), among which 50% are inter-XLs. We also identified several inter-XL specific to each condition: 4 RuvBL2-SPAG1 XLs in the 25:1 condition; 1 RuvBL1-RuvBL2 XL and 3 RuvBL2-SPAG1 XL peptide in the 100:1 condition; and 3 more RuvBL1-RuvBL2 inter-XLs at 400:1 DSBU:R2SP. These results highlight the complementarity of XL interactions captured at low and high reagent concentrations, as already reported in a proteome-wide study^35^. Finally, when combining the results from all three datasets, we identified 127 unique XLs (see Supplementary Table 3) that correspond to 57 inter-XL (41 R1-R2, 5 R1-SPAG1, 11 R2-SPAG1, 45% of total XL peptides) and 70 intra-XL peptides (26 R1-R1, 21 R2R2, 23 SPAG1-SPAG1, 55% of total XL peptides). In particular, our results suggest that the RPAP3 domain of SPAG1 interacts with both the DI domain of RuvBL1 and the DIII domain of RuvBL2. Additionally, the TPR3 domain of SPAG1 appears to be in close proximity to the DIII domain of RuvBL1. We also plotted interactions between R1 and R2 we identified in our analysis onto the available X-ray structure of the R1R2 complex (PDB:2XSZ), and observed excellent agreement between measured and structure-predicted XLs, with 93% (11 intra-XLs, 14 inter-XLs) satisfying the maximal Cα-Cα distances of 30 Å (accepted range for DSBU). Taken together, the R2SP example showcases how dMP can facilitate structural biology studies by rapidly screening cross-linking reaction conditions and reagents. Given that many complexes in biology are transient and/or difficult to isolate and stabilize for structural characterization, we expect that dMP-enabled XL reaction condition screening and optimization will accelerate progress in this area.

## Discussion

We report here on the development of a denaturing MP (dMP)-based protocol for rapid and reproducible screening of XL reaction conditions. The dMP approach consists of a fast and efficient denaturing protocol that results in >95% denaturation of the chemically cross-linked sample within 5 min without altering the quality of the MP measurements or affecting the cross-links. This step is followed by the MP data acquisition that captures the mass distribution of the cross-linked species in the matter of minutes. The mass resolution of dMP is sufficient to capture not only the final complex, but all the sub-complexes that form en route to the ultimate one. Furthermore, the method yields information about the relative quantities of the multimers that form during the cross-linking reactions, thus enabling more precise reaction monitoring and quantification. Overall, when compared to SDS-PAGE, which is a preferred method for XL reaction quality control, dMP provides more accurate mass estimations across a broader mass range (30 kDa to 5 MDa), and with significantly higher sensitivity, thus enabling unambiguous detection of even low-abundance species. In addition, dMP is much faster (20 XL reaction conditions can be screened in triplicate in 1 hour, compared to several hours (2.5 to 20 h) needed per single run of precasted or in-house made SDS-PAGE gels^7^), and uses 20 to 100-times less material (<1 ng, 0.1 pmol at 5 nM as routine conditions per triplicate) than SDS-PAGE (1–5 µg, single run^7,36^). Taken together, dMP strategy provides an unmatched increase in the speed and quality of screening of XL conditions, as well as enables identification and relative quantification of all coexisting cross-linked species with a single molecule sensitivity.

As cross-linking reactions are widely used in structural biology to stabilize complexes prior to their biophysical and structural characterization, we expect that dMP will significantly expand the number and type of samples that can be analyzed, thanks to its high sensitivity, high speed, and high mass-accuracy. We used dMP to rapidly screen multiple XL reaction conditions and XL reagents, and used two new type of metrics to assess XL efficiencies/stabilizations (Eff_XL_, SF_XL_). We showed that longer and more flexible XL reagents, such as DSBU, facilitate cross-linking more effectively compared to less flexible and smaller DSAU and PhoX^37–39^. Conversely, less flexible XL reagents with smaller sized spacer arms lead to stabilization of sub-complexes instead of intact complexes/oligomers. In addition, we showed that dMP-based method for XL reaction condition screening, rapidly identified optimal conditions for cross-linking of R2SP, a protein complex that has eluded structural characterization. Using dMP in combination with XL-MS, yielded new insights into inter-subunit contacts between components of R2SP, including a previously unknown interface between RPAP3 domain of SPAG1 and DI domain of RuvBL1 and the DIII domain of RuvBL2, as well as the TPR3 domain of SPAG1 and DIII domain of RuvBL1. Although these insights remain to be validated using orthogonal strategies, they illustrate the power of dMP to accelerate XL-MS and cryo-EM workflows, and illuminate previously inaccessible features of macromolecular complexes.

## METHODS

### R2SP expression and purification

RuvBL1ΔT127-E233 (R1ΔDII, further named R1) carries an N-terminal 6x His-tag followed by thrombin cleavage site, while RuvBL2ΔE134-E237 (R2ΔDII, further named R2) carries an C-terminal Flag+FH8 tag preceded by HRV-3C cleavage site (Flag was used for detection)^40^. The RuvBL1ΔT127-E233- RuvBL2 ΔE134-E237 complex was expressed in *Escherichia coli* (DE3) (Novagen, 71400), with 100 μM IPTG overnight at 18 °C. The complex was immobilized in a 5 ml HistrapTM HP (GE Healthcare) and eluted with 300 mM imidazole. Peak fractions collected from the HisTrap were incubated with 5 mM CaCl2 during 1 h and loaded onto an HiPrepTM Octyl FF 16/10 column (GE Healthcare). Bound proteins were eluted using 5 mM EDTA. To remove the FLAG_FH8 tag the collected samples were incubated 18 h at 4 °C with 1% (w/w) HRV-3C protease (Thermo Fisher Scientific). A final Superose 6 equilibrated in buffer 20 mM Tris–HCl pH 8.0, 150 mM NaCl, 5% glycerol, 2 mM MgCl2 and 0.5 mM TCEP, was used to separate a stable dodecameric peak, from the HRV3C protease and cleaved tags. The pooled dodecamer was concentrated to 37.75 mg/ml using a 30 kDa Cut-off Amicon Ultra centrifugal filter (Millipore).

SPAG1(622-926) (SPAG1) carries an C-terminal Flag-tag without a cleavage site, while PIH1D2(231-315) (PIH1D2) has a C-terminal StrepTag II preceded by a Human Rhino Virus 3 C cleavage site (HRV-3C). SPAG1(622-926)/PIH1D2(231-315) were co-expressed in *Escherichia coli* (DE3*) (Novagen, 71400), with 50 µM IPTG overnight at 18 °C in a New Brunswick™ (Innova®) 44 R Shaker at 150 rpm. The SP_mini complex was immobilized in a 5 ml StrepTactin XT HC (IBA life sciences), and eluted with 50 mM Biotin. Peak fractions collected from the StrepTactin XT were Injected in a Superdex 200 16/60 XK equilibrated in Buffer 20 mM Hepes pH 8, 300 mM NaCl, 0.5 mM TCEP, allowing the isolation of a heterodimer. Collected fractions from the main peak were diluted to reduce the concentration of NaCl to 50 mM, and further polished in ResourceTM Q (GE), and eluted with a linear gradient, allowing the separation of a major peak corresponding to the intact complex (w/o degradation) at approximately 170 mM NaCl. Collected peak fractions were supplemented with 20 mM imidazole and tag removal was performed by incubating 18 h at 4 °C with 1% (w/w) HRV-3C protease (Thermo Fisher Scientific). Digested sample was injected in a 5 ml StrepTactin XT in tandem with 1 ml HisTrap. The collected flow was concentrated to 14.7 mg/ml using a 3 kDa Cut-off Amicon Ultra centrifugal filter (Millipore). All purification steps were carried out at room temperature and were monitored by NuPAGE Bis-Tris gels (Invitrogen, NP0302).

### Sample preparation

Bovine Serum Albumin (BSA, Sigma, Saint-Louis, USA), Alcohol dehydrogenase from baker’s yeast (ADH, Sigma, Saint-Louis, USA) and L-Glutamate dehydrogenase from bovine liver (GLDH, Sigma, Saint-Louis, USA) were diluted to 1 mg/mL in Gibco^TM^ phosphate buffer saline (PBS, Life technologies Corporation, NY, USA), pH 7.4. Human 20 S proteasome (20 S, South Bay Bio, San Jose, USA) was diluted to 1 mg/mL in 50 mM HEPES, 100 mM NaCl, pH 7.4 prior to cross-linking (see Supplementary Table 4 for composition and molecular weights of different assemblies).

R2SP complex was formed by mixing pure RuvBL1(ΔT127-E233)/RuvBL2(ΔE134-E237) with excess pure SPAG1(622-926)/PIH1D2(231-315) complex at a ratio of 1: 4 (considering RuvBLs dodecameric, and SPAG1/PIH1D2 heterodimeric) over night at 4 °C. Formed R2SP complex was separated from free excess SPAG1/PIH1D2 using a Superose 6 16/60 XK (GE Healthcare) previously equilibrated in 20 mM Hepes pH 8, 150 mM NaCl, 2 mM MgCl2, and 0.5 mM TCEP. The eluted peak was concentrated to 7.5 mg/ml using a 3 kDa Cut-off Amicon Ultra centrifugal filter (Millipore).It was diluted to 1 mg/mL in 20 mM Hepes, 150 mM NaCl, pH 8^31^ prior to cross-linking.

### Cross-linking reactions

For NHS-ester-based cross-linking (XL) reactions, all aliquots of XL reagents were freshly diluted in anhydrous DMSO (Invitrogen™ by Thermo Fisher Scientific, Rockford, IL, USA). Following reagents were used: PhoX (Disuccinimidyl Phenyl Phosphonic Acid, Bruker); DSAU (Disuccinimidyl diacetic urea, CF Plus Chemicals, Brno-Řečkovice, Czech Republic); DSSO (Disuccinimidyl sulfoxide, Thermo Fisher Scientific, Rockford, IL, USA); DSBU (Disuccinimidyl dibutyric urea, CF Plus Chemicals, Brno-Řečkovice, Czech Republic). BSA, ADH, GLDH and 20S samples were each split in six aliquots and incubated with 25, 100, or 400 molar excess of each reagent. All samples were additionally cross-linked with 800 and 100 molar excesses of DSBU. For R2SP cross-linking, stock solution was split into 20 aliquots subsequently reacted with PhoX, DSAU, DSSO, DSBU at molar excesses of 25, 50, 100, 200, 400.

For formaldehyde cross-linking, GLDH and 20 S proteasome at 1 mg/mL were incubated by adding a formaldehyde 37% stock solution (Merck KGaA, Darmstadt, Germany) diluted to 0.05%, 0.1%, 0.2%, 0.5% and 1% (vol/vol final) for 20 min at room temperature^41^.

All XL reactions were carried for all samples at room temperature (20 °C) for 45 min, and quenched with Tris HCl (15 mM final concentration) for 20 min. An aliquot of each non-XL control and XL sample was kept for SDS-PAGE migration.

### SDS-PAGE separation of cross-linked samples

All cross-linked proteins and complexes were migrated on in-house 12% acrylamide denaturing SDS-PAGE gels (1.5 mm thickness). Volume corresponding to 1 µg of each XL sample (and non-XL controls) was diluted (1:1) with 2x concentrated Læmmli buffer (4% SDS, 20% glycerol, 10% 2-mercaptoethanol, 0.01% bromphenol blue and 0.125 M Tris HCl) and incubated 5 min at 95 °C. After sample loading, gels were migrated at 50 V for 20 min, 100 V until the 2/3 of the gel and 120 V until the end. After migration, gels were fixated for 20 min (3% phosphoric acid, 50% ethanol), washed 3 × 20 min with milli-Q water and stained overnight with Coomasie Brillant Blue (G250, Sigma, Saint-Louis, USA). They were finally rinced 3 × 20 min with milli-Q water.

### Mass photometry measurements

MP measurements were performed with a TWO^MP^ (Refeyn Ltd, Oxford, UK) at room temperature (18 °C). Microscope slides (24 × 50 mm, 170 ± 5 µm, No. 1.5H, Paul Marienfeld GmbH & Co. KG, Germany) were cleaned with milli-Q water, isopropanol, milli-Q water and dried with a clean nitrogen stream. Six-well reusable silicone gaskets (CultureWell^TM^, 50–3 mm DIA x 1 mm Depth, 3–10 µL, Grace Bio-Labs, Inc., Oregon, USA) were carefully cut and assembled on the cover slide center. After being placed in the mass photometer and before each acquisition, an 18 µL droplet of PBS was put in a well to enable focusing on the glass surface.

#### Contrast-to-mass calibration

To allow MP mass measurements, contrast-to-mass calibration was performed twice a day by measuring a mix of Bovine Serum Albumin (66 kDa), Bevacizumab (149 kDa), and Glutamate Dehydrogenase (318 kDa) in PBS buffer, pH 7.4. The distributions of scattering events (given as contrast) were Gaussian-fitted using DiscoverMP (Supplementary Fig. 10a). Contrasts values are converted into masses using linear relation between the contrast and the mass of the binding object. Calibrations were accepted for R^2^ > 0.995 (Supplementary Fig. 10b).

#### Native MP (nMP)

Samples were first diluted with their native buffer to 100–400 nM. Finally, 2 µL of the stock solution are finally drop-diluted and carefully mixed to 10-40 nM in a 18 µL PBS droplet^17^. Three movies of 3000 frames were recorded (60 s) for each sample using the AcquireMP software (Refeyn Ltd, Oxford, UK).

#### Denaturing MP (dMP)

Denaturing MP experiments were carried out by incubating first the samples to a protein concentration of 100–400 nM in 5.4 M Urea (Sigma, Saint-Louis, USA) or 6 M Guanidine (Sigma, Saint-Louis, USA). For non-crosslinked samples incubation times evaluated ranged from 5 min to 16 hours at room temperature (18 °C). After incubation and right before MP measurements, 2 µL of the solution were quickly drop-diluted^11^ in an 18 µL PBS droplet to 10–40 nM. All measurements were done immediately following the droplet dilution. For the final optimized dMP protocol, denaturation was done in Urea 5.4 M for 5 min.

#### MP Data processing

Data were processed using the DiscoverMP software (Refeyn Ltd, Oxford, UK). Obtained distribution histograms represent the number of counts per contrast value (or per mass after calibration). To obtain the average masses, peak width and number of counts for each mass distribution, a Gaussian fitting was performed by integrating each distributions at its half-height. Relative amounts of each oligomer were calculated using the number of counts under the Gaussian fit curve of each distribution. For figures, Kernel Density Estimate (KDE) was applied to transform the histogram into a curve.

### Calculation of the total inter-protein cross-linking reaction efficiency (Eff_XL_)

Total inter-XL efficiency (1) was calculated using number of counts after Gaussian fitting of each oligomeric state distribution (example of calculation in Supplementary Table 5). This value represent the efficiency of XL reaction to form inter-protein interactions, i.e. all oligomeric states >1 remaining after denaturation. Inter-XL efficiency does not discriminate between specific interactions and non-specific aggregation.

1 $${{{{{\rm{Total}}}}}}\,{{{{{\rm{XL}}}}}}\,{{{{{\rm{efficiency}}}}}}=\overline{\left(\frac{\sum {s}_{oligomers > 1}}{\sum s}\right) \,*\, 100 \,=\,\%}\pm {{{{{\rm{SD}}}}}}$$

Equation 1. $\sum {S}_{{oligomers} > 1}$ is the sum of all populations with oligomeric states >1; $\sum {S}$ is the sum of all counts for masses > 30 kDa

### Calculation of the specific XL factor (SF_XL_)

Specific factor is defined as the specific/intended product of the XL reaction, i.e. the stabilization of native complex. We first use non-XL native measurements as a reference to obtain the proportion represented by the complex to be XL-stabilized in the sample. Then, we similarly calculated the proportion of this complex among total counts of the cross-linked denatured sample. Using these two values, the complex stabilization factor can be calculated (example of calculation in Supplementary Table 6). This value expresses the amount of native complex that could effectively be XL-stabilized in XL samples (2). A factor value of 1 correspond to the stabilization of all the native complex after XL reaction. Value > 1 expresses an enrichment of the complex upon XL reaction. Stabilization factor should be ideally ≥ 1.

2 $${{{{{\rm{Specific}}}}}}\,{{{{{\rm{XL}}}}}}\,{{{{{\rm{factor}}}}}}=\overline{\left(\frac{{S}_{complex}(XL)}{\sum S(XL)}/\frac{{S}_{complex}(CT)}{\sum S(CT)}\right)}$$

Equation 2. ${{{{{{\boldsymbol{S}}}}}}}_{{{{{{\boldsymbol{complex}}}}}}}$ is the integrated number of counts corresponding to the complex in both the cross-linked dMP XL sample $\left({{{{{\boldsymbol{XL}}}}}}\right)$ and nMP non-XL sample $\left({{{{{\boldsymbol{CT}}}}}}\right)$; $\sum S$ is the sum of all integrated populations (monomer included) in the cross-linked dMP XL sample $\left({{{{{\boldsymbol{XL}}}}}}\right)$ or nMP non-XL reference sample $\left({{{{{\boldsymbol{CT}}}}}}\right)$.

### Cross-linking mass spectrometry

R2SP complex was cross-linked in triplicates at each 25, 100, 400 molar excesses of DSBU (45 min, 18 °C), before quenching reaction with 15 mM Tris HCl (20 min). Samples were reduced by adding DTT to a final concentration of 5 mM and incubation at 60 °C for 30 min. The alkylation was done by adding Iodoacetamide to a final concentration of 15 mM (1-hour incubation step in the dark). Samples were then processed with overnight digestion with Trypsin/Lys-C (Promega, Madison, USA) at a 50:1 substrate:enzyme ratio (w/w) at 37 °C overnight. The digestions were finally quenched with 1% TFA.

Peptides were cleaned up by using the AssayMAP Bravo platform (Agilent Technologies; Santa Clara, California) with 5 μL C18 cartridges (Agilent). Cartridges were primed with 100 μl 0.1% TFA in 80% ACN and equilibrated with 50 μl 0.1% TFA in H2O. 180 μl of digested peptides diluted in equilibration buffer were loaded on the cartridges and washed with 50 μl equilibration buffer. Peptides were eluted with 50 μl 0.1% TFA in 80% ACN and stored at −80 °C prior to the mass spectrometry analysis. Cross-linked peptides were dried in a SpeedVac concentrator and resuspended in 2% ACN/0.1% formic acid.

NanoLC-MS/MS analysis was performed using a nanoAcquity UPLC (Waters, Milford, USA) hyphenated to a Q Exactive HF-X mass spectrometer (Thermo Fisher Scientific, Bremen, Germany) equipped with a nanoSpray source. After trapping on a NanoEase M/Z Symmetry pre-column (C18, 100 Å, 5 μm, 180 μm × 20 mm; Waters), samples were separated on a NanoEase M/Z BEH column (C18, 130 Å, 1.7 μm, 75 μm × 250 mm; Waters) maintained at 60 °C. A gradient of 102 min was applied: mobile phases A (0.1% v/v formic acid in H2O) and B (0.1% v/v formic acid in ACN). The following conditions were applied: 3% B for 3 min, 3–40% B for 90 min, 40–90% B for 1 min, 90% B for 5 min, 90–1% B for 2 min and finally 1% B maintained for 2 min (flow rate of 350 nl/min). Acquisition in Data Dependant Acquisition mode (Top 10 precursor ions) was done using following parameters: MS resolution of 120.000 (AGC target 3e6), MS/MS resolution of 30.000 (AGC target of 2e5), 3–7 charge states enabled, HCD stepped collision energy (27, 30, 33% normalized collision energy). Raw data were directly processed with Thermo Proteome Discoverer 2.5.0.400 (Thermo Scientific) using the XlinkX node for identification of crosslinks and the Sequest HT node for the identification of linear peptides. For linear peptides identification a database containing R2SP sequences and common contaminants was used. As R2SP complex purity was high, a reduced database containing R2SP subunit sequences (Supplementary Fig. 11) was used for XL identification. For both linear and cross-linked peptides searches, Cystein carbamidomethylation was set as fixed modification. Methionine oxidation, N-term acetylation, tris-quenched mono-links and water-quenched mono-links were set as dynamic modifications. Trypsin was set as the cleavage enzymes with minimal length of 7 amino acids, 2 (linear peptides) and 3 (cross-linked peptides) missed cleavages were allowed, respectively for proteomics and XL identifications. Mass accuracies for both XL and linear peptides seach were set to 10 ppm for MS1 and 0.05 Da for MS2. To increase confidence, identification were only accepted for Maximal XlinkX scores > 40 and Δ*score* > 4. A 1% false discovery rate was applied for both linear and XL-peptides, and XLs were further manually curated. Out of the three replicates performed for each molar excess of DSBU, unique cross-link sites were validated only when present in at least 2 out of 3 replicates.

Data were visualized using xiVIEW webserver (www.xiview.org), to produce a circular interaction network and represent XL sites on protein sequences^42,43^. Finally, validated cross–links (2/3 file threshold) were plotted on 2XSZ X-ray diffraction structure^31^ corresponding to the R1R2 complex. The PyMol Molecular Graphics System (version 2.5.4, Schrödinger, LLC) was used as well as xiVIEW server to visualize and measure XLs Cα-Cα distances on the structure. Corresponding distances were only validated if within ≤30 Å threshold.

### Reporting summary

Further information on research design is available in the Nature Portfolio Reporting Summary linked to this article.

### Supplementary information

Supplementary Information

Reporting Summary

Peer Review File

### Source data

Source Data

## Data Availability

The XL-MS dataset (R2SP with 25, 100, 400 molar excesses of DSBU) generated in this study, including experimental settings and XL identification results, has been deposited on the ProteomeXchange Consortium via the PRIDE^44^ repository with the dataset identifier PXD042549 (R2SP cross-linking mass spectrometry). The Mass Photometry raw and treated files generated in this study will be made fully available upon request. Source data are provided with this paper.

## References

[1] Piersimoni, L., Kastritis, P. L., Arlt, C. & Sinz, A. Cross-Linking Mass Spectrometry for Investigating Protein Conformations and Protein–Protein Interactions─A Method for All Seasons. *Chem. Rev*. **122**, 7500–7531 (2022). 10.1021/acs.chemrev.1c00786 34797068

[2] Bartolec TK (2023). Cross-linking mass spectrometry discovers, evaluates, and corroborates structures and protein–protein interactions in the human cell. Proc. Natl Acad. Sci. USA.

[3] Schmidt C, Kramer K, Urlaub H (2012). Investigation of protein–RNA interactions by mass spectrometry—Techniques and applications. J. Proteom..

[4] Stützer A (2020). Analysis of protein-DNA interactions in chromatin by UV induced cross-linking and mass spectrometry. Nat. Commun..

[5] Carson FL (2000). Formaldehyde as a fixative for light and electron microscopy. Microsc. Today.

[6] Hoffman EA, Frey BL, Smith LM, Auble DT (2015). Formaldehyde crosslinking: a tool for the study of chromatin complexes *. J. Biol. Chem..

[7] Iacobucci C (2018). A cross-linking/mass spectrometry workflow based on MS-cleavable cross-linkers and the MeroX software for studying protein structures and protein–protein interactions. Nat. Protoc..

[8] Chavez JD (2019). Systems structural biology measurements by in vivo cross-linking with mass spectrometry. Nat. Protoc..

[9] Iacobucci C (2019). First community-wide, comparative cross-linking mass spectrometry study. Anal. Chem..

[10] Asor R, Kukura P (2022). Characterising biomolecular interactions and dynamics with mass photometry. Curr. Opin. Chem. Biol..

[11] Olerinyova A (2021). Mass photometry of membrane proteins. Chem.

[12] Lai S-H, Tamara S, Heck AJR (2021). Single-particle mass analysis of intact ribosomes by mass photometry and Orbitrap-based charge detection mass spectrometry. iScience.

[13] Wu D, Hwang P, Li T, Piszczek G (2022). Rapid characterization of adeno-associated virus (AAV) gene therapy vectors by mass photometry. Gene Ther..

[14] Young G (2018). Quantitative mass imaging of single biological macromolecules. Science.

[15] Young G, Kukura P (2019). Interferometric scattering microscopy. Annu. Rev. Phys. Chem..

[16] Dong J, Maestre D, Conrad-Billroth C, Juffmann T (2021). Fundamental bounds on the precision of iSCAT, COBRI and dark-field microscopy for 3D localization and mass photometry. J. Phys. Appl. Phys..

[17] Wu D, Piszczek G (2021). Standard protocol for mass photometry experiments. Eur. Biophys. J..

[18] Paul SS, Lyons A, Kirchner R, Woodside MT (2022). Quantifying oligomer populations in real time during protein aggregation using single-molecule mass photometry. ACS Nano.

[19] den Boer MA (2022). Comparative analysis of antibodies and heavily glycosylated macromolecular immune complexes by size-exclusion chromatography multi-angle light scattering, native charge detection mass spectrometry, and mass photometry. Anal. Chem..

[20] Sonn-Segev A (2020). Quantifying the heterogeneity of macromolecular machines by mass photometry. Nat. Commun..

[21] Foley EDB, Kushwah MS, Young G, Kukura P (2021). Mass photometry enables label-free tracking and mass measurement of single proteins on lipid bilayers. Nat. Methods.

[22] Niebling, S. et al. Biophysical screening pipeline for cryo-EM grid preparation of membrane proteins. *Front. Mol. Biosci*. **9**10.3389/fmolb.2022.882288(2022). 10.3389/fmolb.2022.882288 PMC9259969 35813810

[23] Ebberink EHTM, Ruisinger A, Nuebel M, Thomann M, Heck AJR (2022). Assessing production variability in empty and filled adeno-associated viruses by single molecule mass analyses. Mol. Ther. - Methods Clin. Dev..

[24] Lim WK, Rösgen J, Englander SW (2009). Urea, but not guanidinium, destabilizes proteins by forming hydrogen bonds to the peptide group. Proc. Natl Acad. Sci..

[25] Das A, Mukhopadhyay C (2009). Urea-Mediated Protein Denaturation: A Consensus View. J. Phys. Chem. B.

[26] Huerta-Viga A, Woutersen S (2013). Protein Denaturation with Guanidinium: A 2D-IR Study. J. Phys. Chem. Lett..

[27] Steigenberger B, Pieters RJ, Heck AJR, Scheltema RA (2019). PhoX: an IMAC-enrichable cross-linking reagent. ACS Cent. Sci..

[28] Kastner B (2008). GraFix: sample preparation for single-particle electron cryomicroscopy. Nat. Methods.

[29] Strauss JD, Wagenknecht T (2013). Structure of glutaraldehyde cross-linked ryanodine receptor. J. Struct. Biol..

[30] Drulyte I (2018). Approaches to altering particle distributions in cryo-electron microscopy sample preparation. Acta Crystallogr. Sect. Struct. Biol..

[31] Gorynia S (2011). Structural and functional insights into a dodecameric molecular machine – The RuvBL1/RuvBL2 complex. J. Struct. Biol..

[32] Maurizy C (2018). The RPAP3-Cterminal domain identifies R2TP-like quaternary chaperones. Nat. Commun..

[33] Seraphim TV (2022). Assembly principles of the human R2TP chaperone complex reveal the presence of R2T and R2P complexes. Structure.

[34] Matzinger M, Mechtler K (2021). Cleavable cross-linkers and mass spectrometry for the ultimate task of profiling protein–protein interaction networks in vivo. J. Proteome Res..

[35] Fürsch J, Kammer K-M, Kreft SG, Beck M, Stengel F (2020). Proteome-wide structural probing of low-abundant protein interactions by cross-linking mass spectrometry. Anal. Chem..

[36] Klykov O (2018). Efficient and robust proteome-wide approaches for cross-linking mass spectrometry. Nat. Protoc..

[37] Chen F, Nielsen S, Zenobi R (2013). Understanding chemical reactivity for homo- and heterobifunctional protein cross-linking agents: Chemical cross-linking efficiency in proteins. J. Mass Spectrom..

[38] Beveridge R, Stadlmann J, Penninger JM, Mechtler K (2020). A synthetic peptide library for benchmarking crosslinking-mass spectrometry search engines for proteins and protein complexes. Nat. Commun..

[39] Ihling CH, Piersimoni L, Kipping M, Sinz A (2021). Cross-linking/mass spectrometry combined with ion mobility on a timsTOF pro instrument for structural proteomics. Anal. Chem..

[40] Dermouche S, Chagot M-E, Manival X, Quinternet M (2021). Optimizing the first TPR domain of the human SPAG1 protein provides insight into the HSP70 and HSP90 binding properties. Biochemistry.

[41] Cong Y (2010). 4.0-Å resolution cryo-EM structure of the mammalian chaperonin TRiC/CCT reveals its unique subunit arrangement. Proc. Natl Acad. Sci. USA.

[42] Combe CW, Fischer L, Rappsilber J (2015). xiNET: cross-link network maps with residue resolution. Mol. Cell. Proteom..

[43] Graham, M., Combe, C., Kolbowski, L. & Rappsilber, J. xiView: A common platform for the downstream analysis of Crosslinking Mass Spectrometry data. Preprint at 10.1101/561829 (2019).

[44] Perez-Riverol Y (2022). The PRIDE database resources in 2022: a hub for mass spectrometry-based proteomics evidences. Nucleic Acids Res..

