# Supplementary material for: Denaturing mass photometry for rapid optimization of chemical protein-protein cross-linking reactions
Source: Nat Commun. 2024 Apr 25;15:3516. doi: 10.1038/s41467-024-47732-4 (PMC11045720; doi:10.1038/s41467-024-47732-4)
Supplement: Supplementary file 1 — Supplementary Information [file 41467_2024_47732_MOESM1_ESM.pdf]

SUPPLEMENTARY INFORMATION

Supplementary Methods

Native mass spectrometry.

R2SP samples were desalted, and buffer exchanged for ammonium acetate 200 mM, pH 7.8 using 7 KDa cutoff Zeba microcentrifuge gel filtration columns (Thermo Fisher Scientific, Rockford, IL, USA). Protein concentrations were determined by UV absorbance using a NanoDrop 2000 spectrophotometer (Thermo Fisher Scientific, France).

Mass spectrometry experiments were carried out on an electrospray time-of-flight mass spectrometer (LCT, Waters, Manchester, UK), coupled with an automated chip-based nanoelectrospray source (Triversa Nanomate, Advion Biosciences, Ithaca, U.S.A.), in the positive ion mode. A first external calibration was performed in denaturing conditions with using the multiply charged ions produced horse heart myoglobin solution diluted to 2 µM in water/acetonitrile/formic acid (50/50/1) using standard interface tuning parameter (V_c of 40 V; $P_{interface}$, 2.1 mbar). For native MS experiments, an additional external calibration was performed using singly charged ions produced by a 2 mg/mL solution of cesium iodide in 2-propanol/water (1/1). For R2SP complex, following MS tuning were used: V_c of 80 V, $P_{interface}$ of 6 mbar. Native data interpretation was performed using MassLynx 4.1 (Waters, Manchester, UK).

Supplementary Figures.

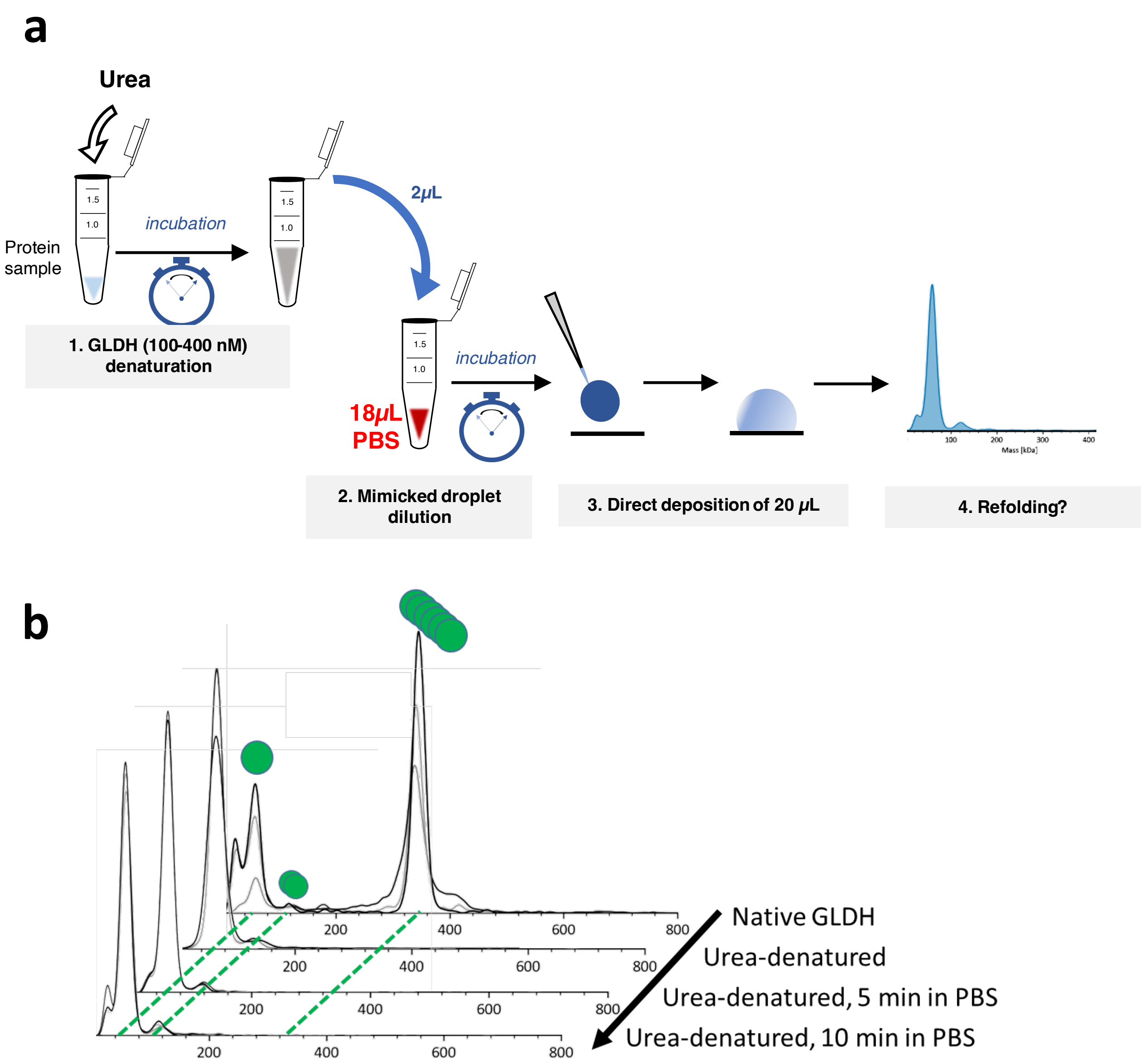

Supplementary Figure 1. GLDH refolding study results mimicking a PBS droplet dilution. a GLDH has been first denatured, then diluted to the tenth in a PBS tube. After 0 min, 5 min and 10 min this solution was directly analyzed in MP using the buffer-free focusing mode. **b** MP profiles of native GLDH, GLDH right after denaturation and after 5 and 10 min mimicked droplet-dilution in PBS.

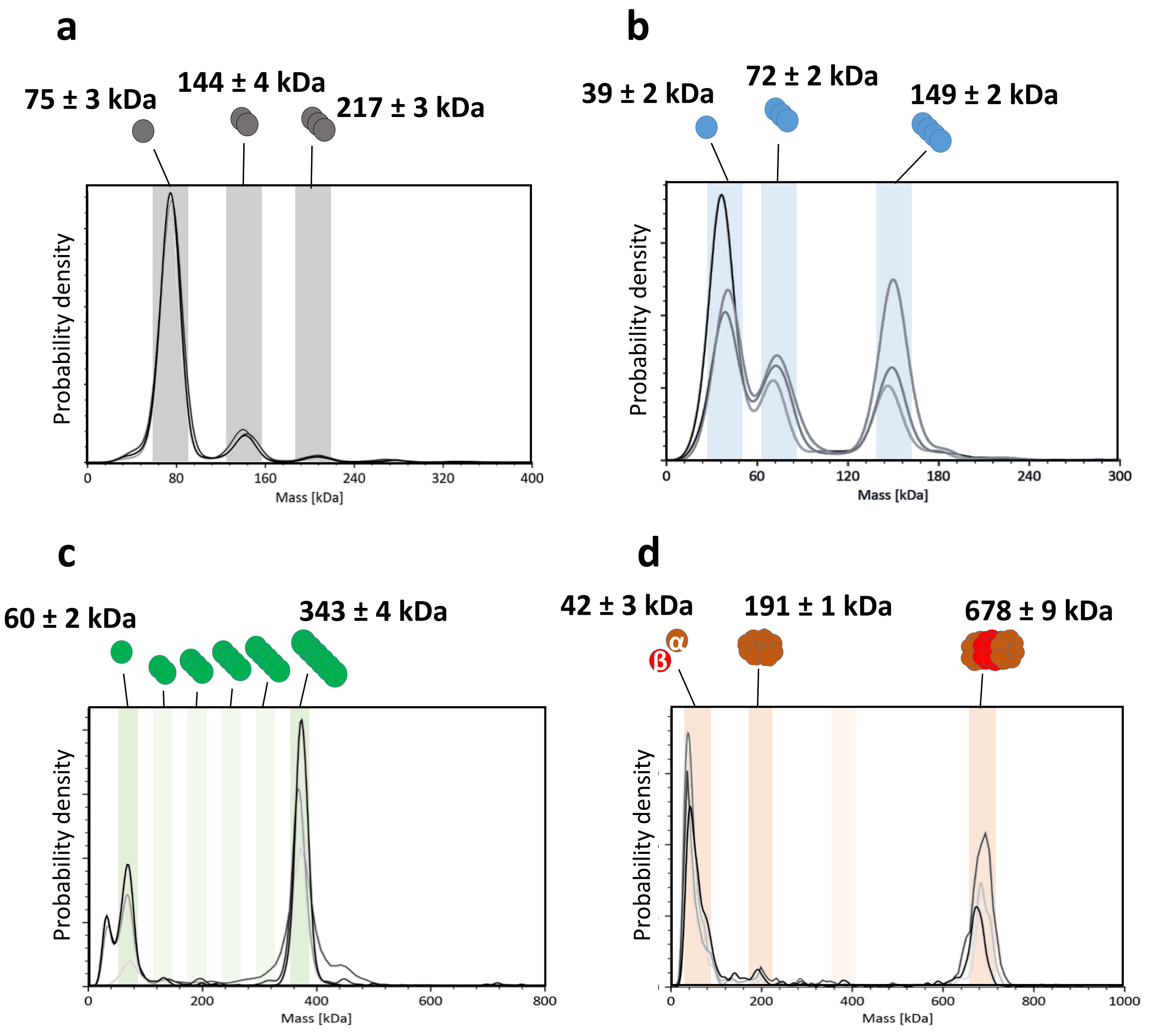

Supplementary Figure 2. Native mass photometry measurements (mean ± SD) highlighting the identified oligomeric populations. **a** BSA, **b** ADH, **c** GLDH and **d** 20S proteasome. Standard deviations of measured masses come from measurement replicates which are shown as overlapping density curves (n=3).

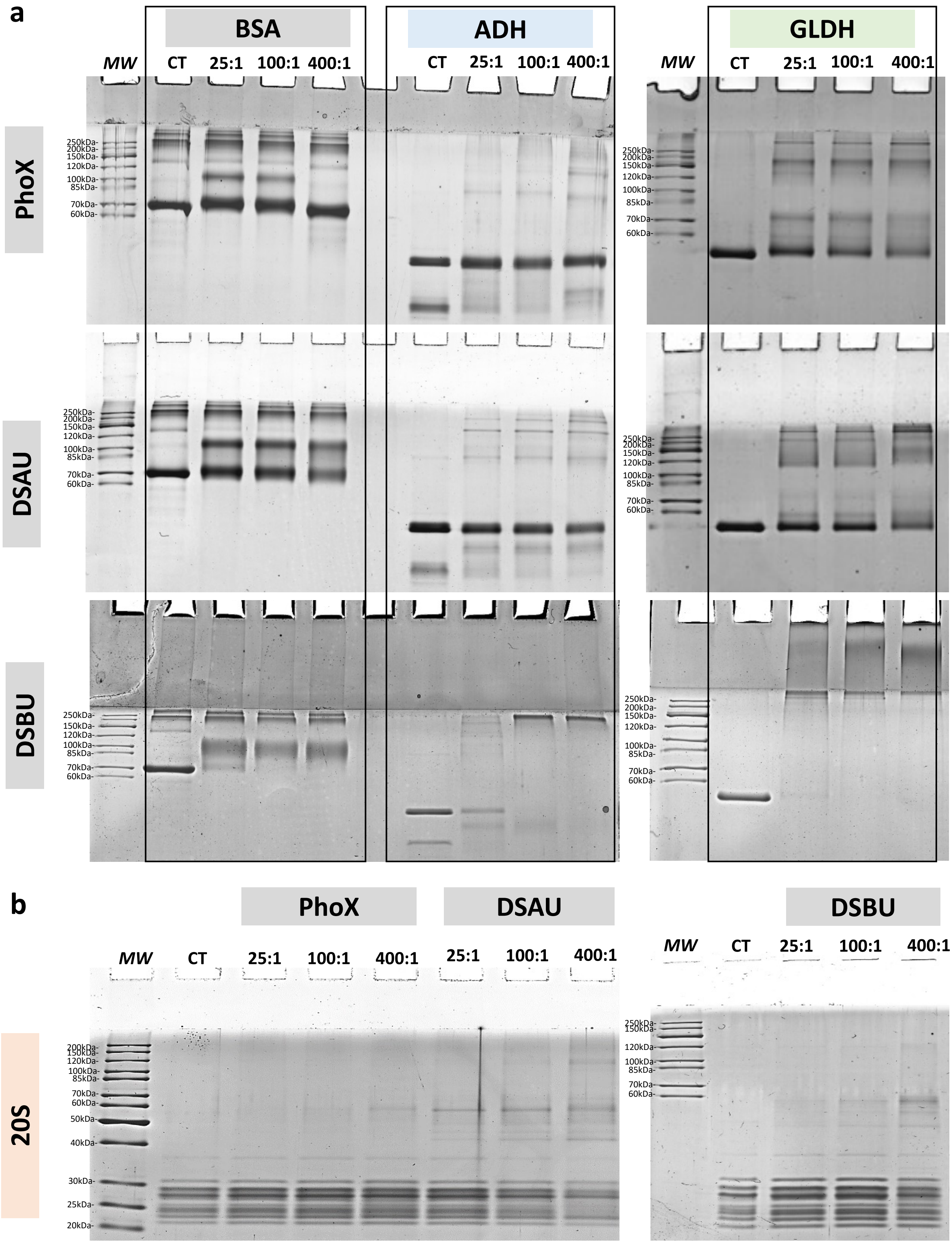

Supplementary Figure 3. SDS-PAGE migrations of **a** BSA, ADH, GLDH cross-linked with 25, 100, 400 molar excesses of PhoX, DSAU, DSBU**. b** 20S proteasome cross-linked with 25, 100, 400 molar excesses of PhoX, DSAU, DSBU.

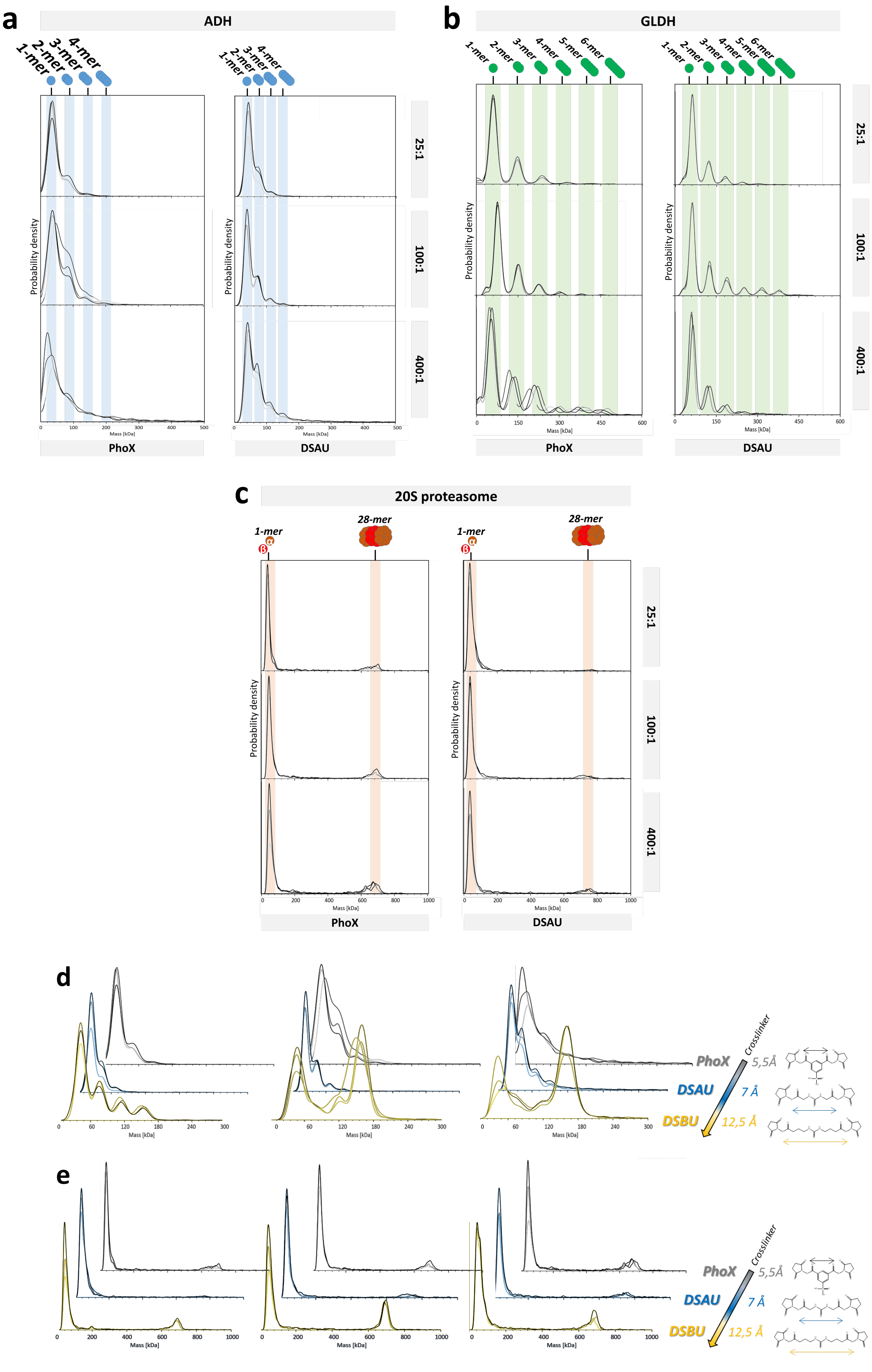
a
ADH
1-mer
2-mer
3-mer
4-mer
Probability density
25:1
100:1
400:1
Mass [kDa]
PhoX
DSAU
b
GLDH
1-mer
2-mer
3-mer
4-mer
5-mer
6-mer
PhoX
DSAU
c
20S proteasome
1-mer
28-mer
PhoX
DSAU
d
e
Crosslinker
PhoX
5,5Å
DSAU
7 Å
DSBU
12,5 Å

Supplementary Figure 4. Additional dMP measurements carried after XL with PhoX and DSBU. a ADH reacted with 25, 100, 400 molar excesses of PhoX and DSAU, **b** GLDH reacted with 25, 100, 400 molar excesses of PhoX and DSAU, **c** 20S proteasome reacted with 25, 100, 400 molar excesses of PhoX and DSAU. Effect of cross-linking reagent on oligomeric states stabilized for **d.** ADH and **e.** 20S proteasome. Measurement replicates are shown as overlapping density curves (n=3).

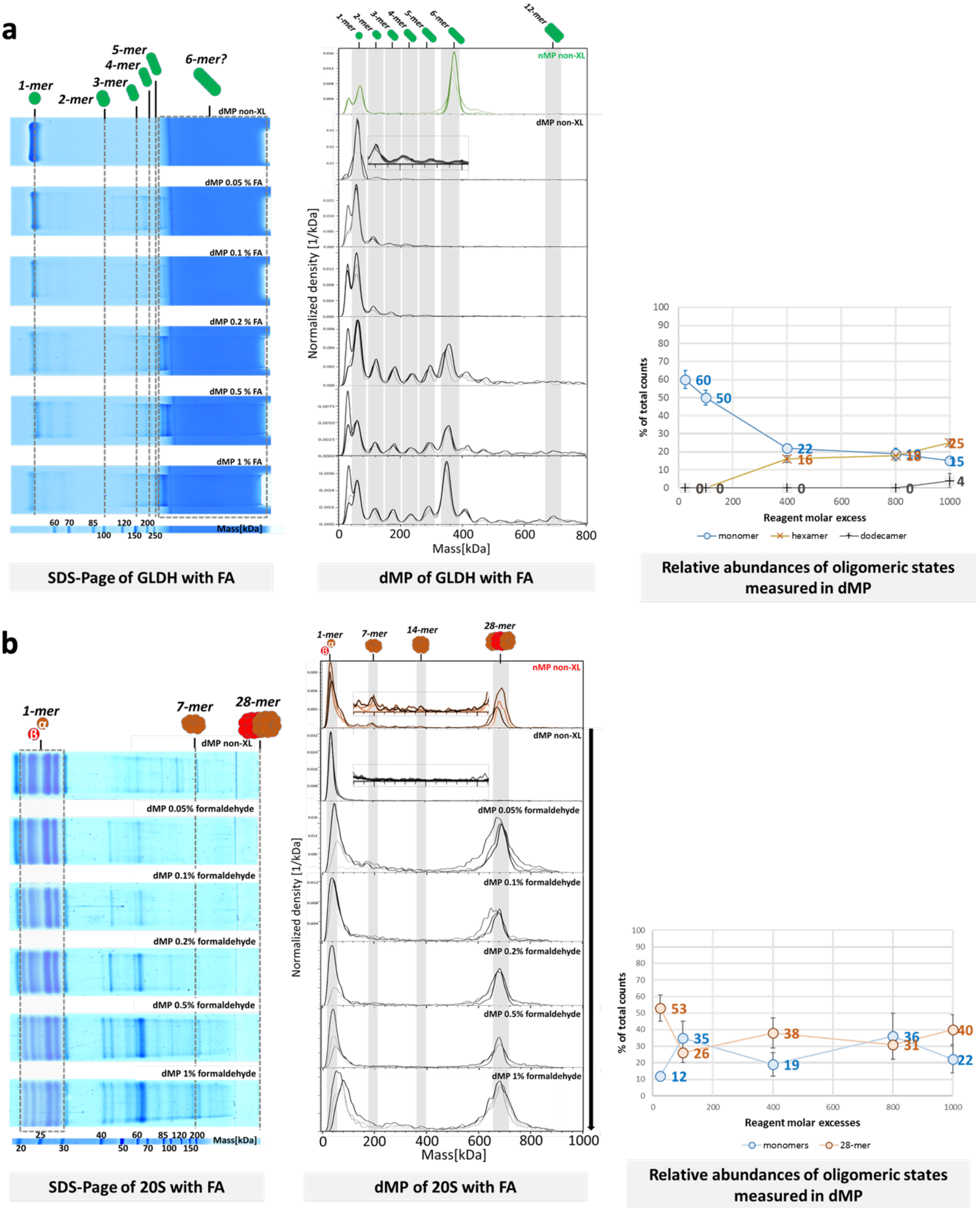

Supplementary Figure 5. Application of dMP to Formaldehyde crosslinking. a GLDH and **b** 20S cross-linked with increased percentages of formaldehyde, with Relative abundances of different oligomeric states shown as scatter plots (% mean ± SD, n=3). dMP measurements replicates (n=3) are shown by overlapping density curves in shades of grey.

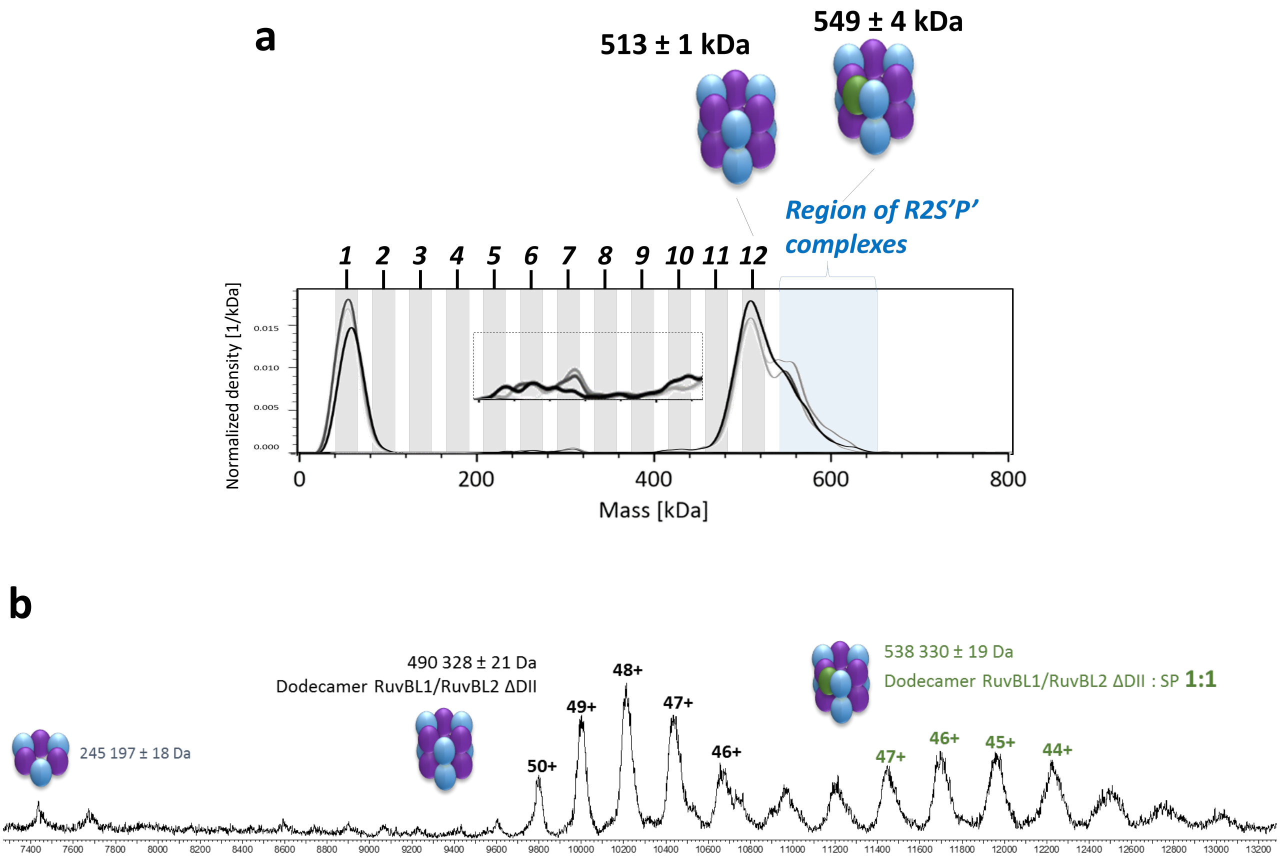

Supplementary Figure 6. Measured mass distribution and oligomeric states identified in R2SP sample. **a** nMP profile of R2SP complex represented as Kernel Density, standard deviation describes the variation of measured mass (peak apex) across replicates (n=3)**. b** Native-MS spectrum of R2SP complex (n=1).

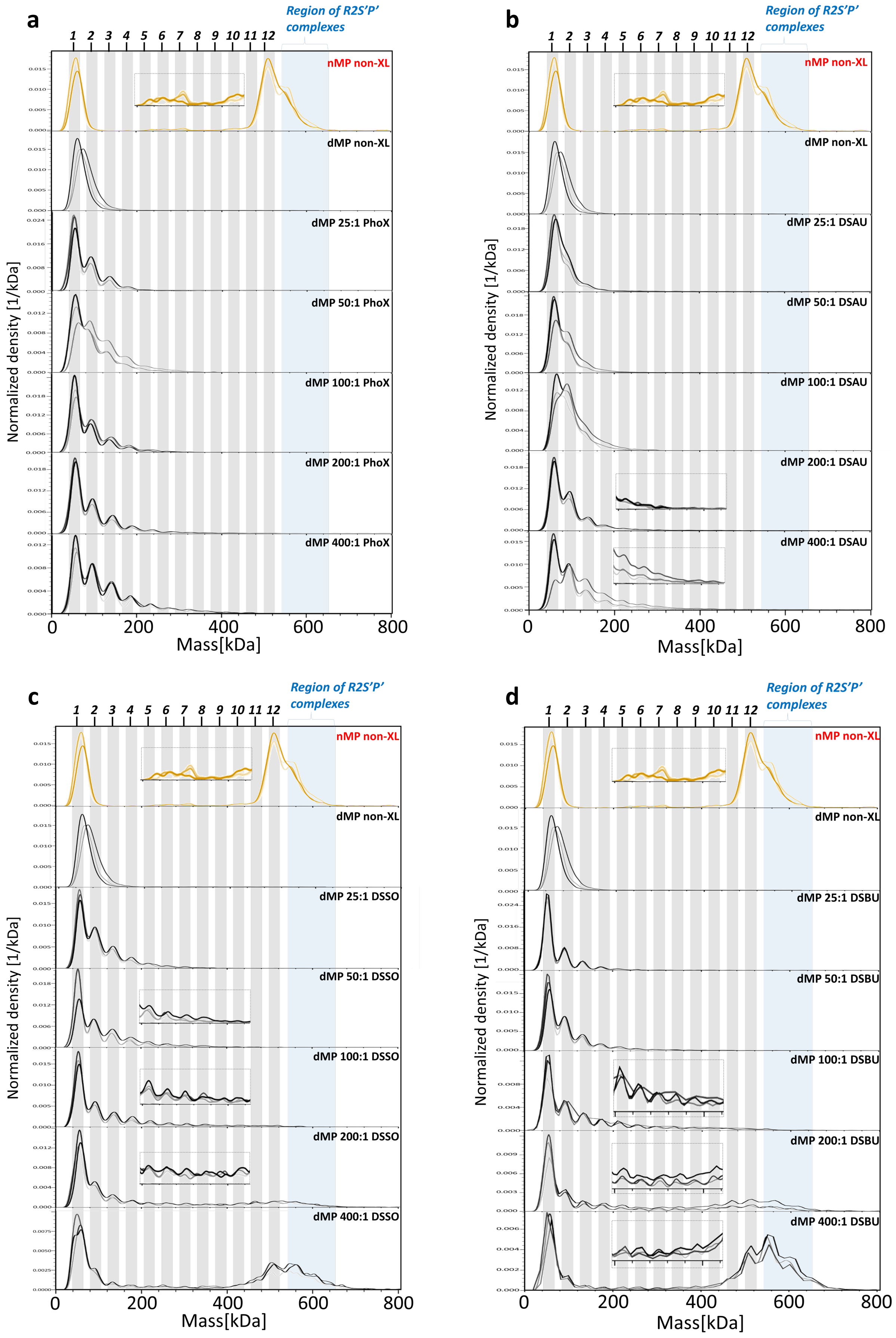

Supplementary Figure 7. Complete dMP profiles of R2SP cross-linked with all 25, 50, 100, 200, 400 molar excesses of **a** PhoX, **b** DSSO, **c** DSAU, **d** DSBU. Measurement replicates (n=3) are shown as overlapping curves.

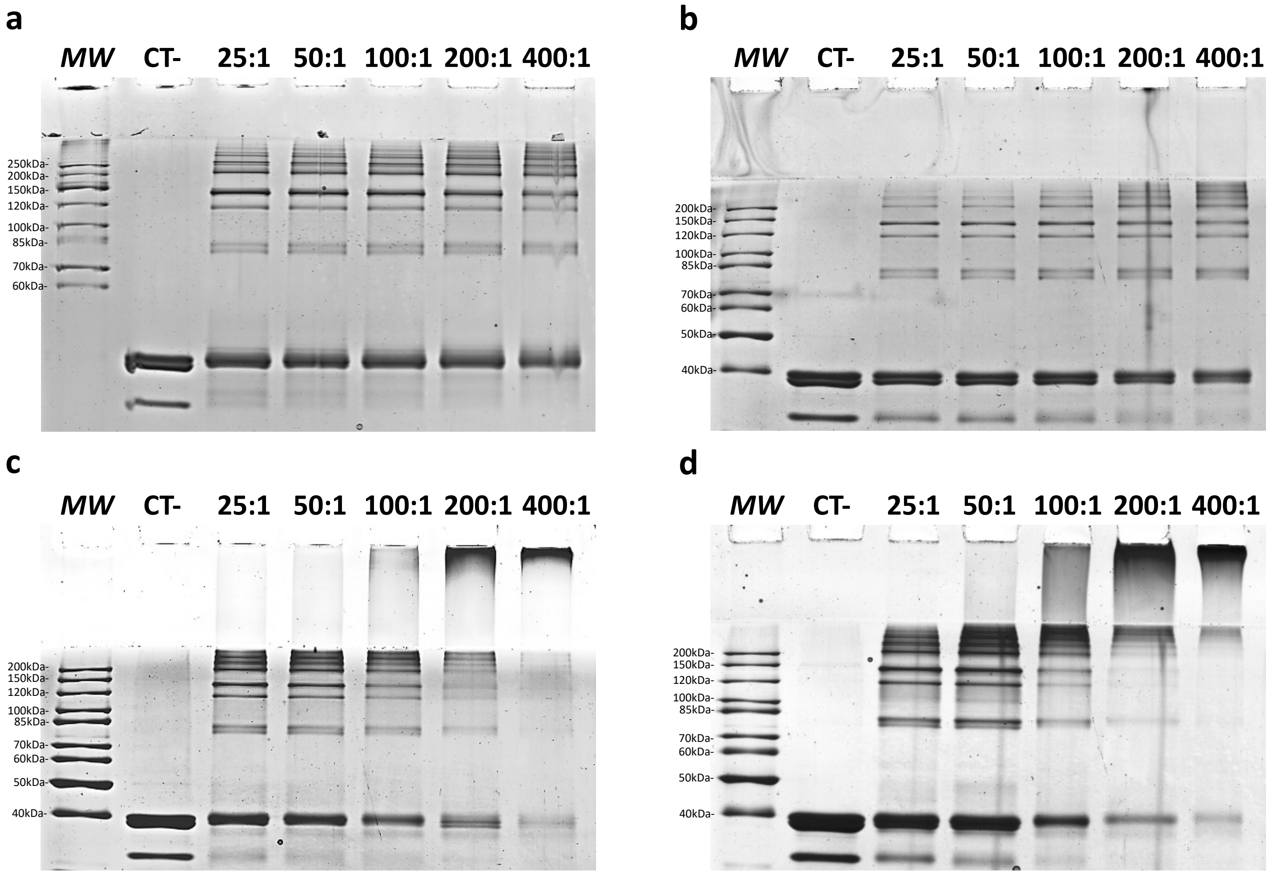

Supplementary Figure 8. SDS-PAGE of R2SP complex cross-linked with 25, 50, 100, 200, 400 molar excesses of **a** PhoX, **b** DSAU, **c** DSSO, **d** DSBU.

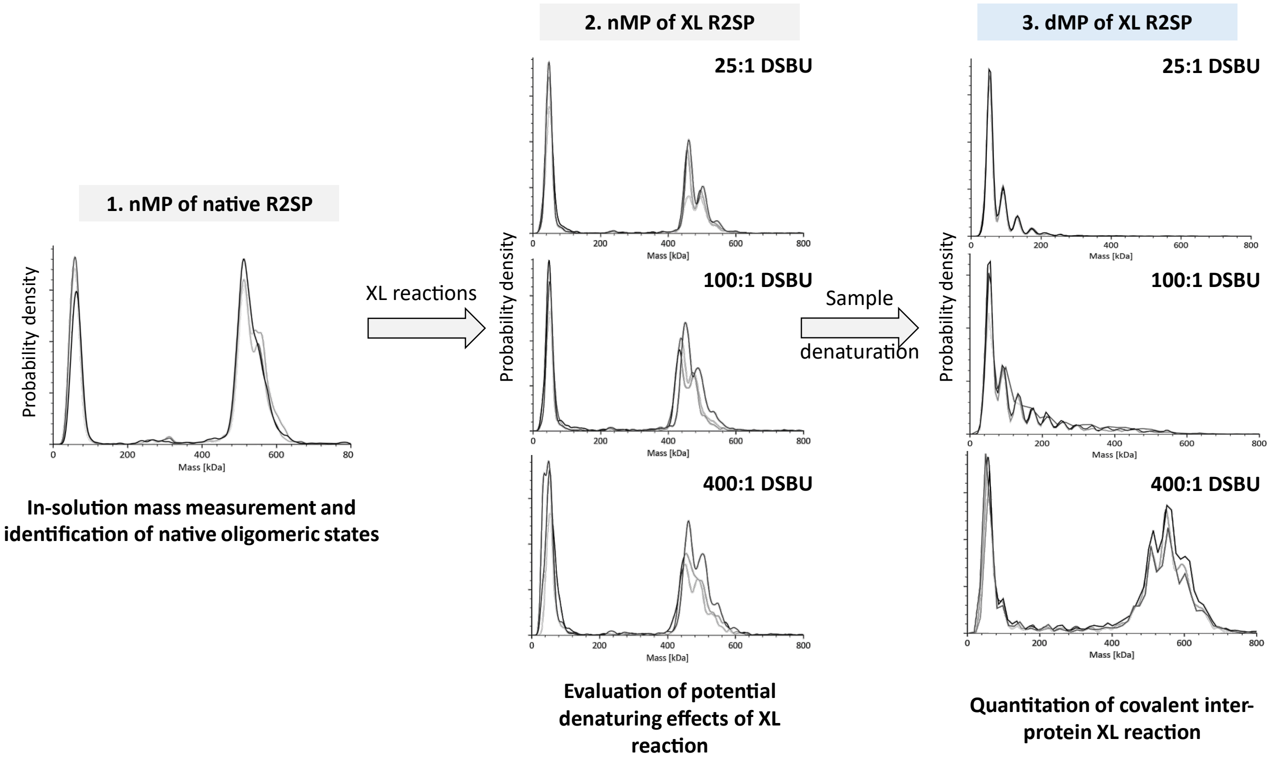

Supplementary Figure 9. Example of nMP and dMP integration to control R2SP complex XL reaction. Concentration shown are 25, 100, 400 molar excesses of DSBU. nMP and dMP measurements replicates (n=3) are shown by overlapping density curves in shades of grey.

a

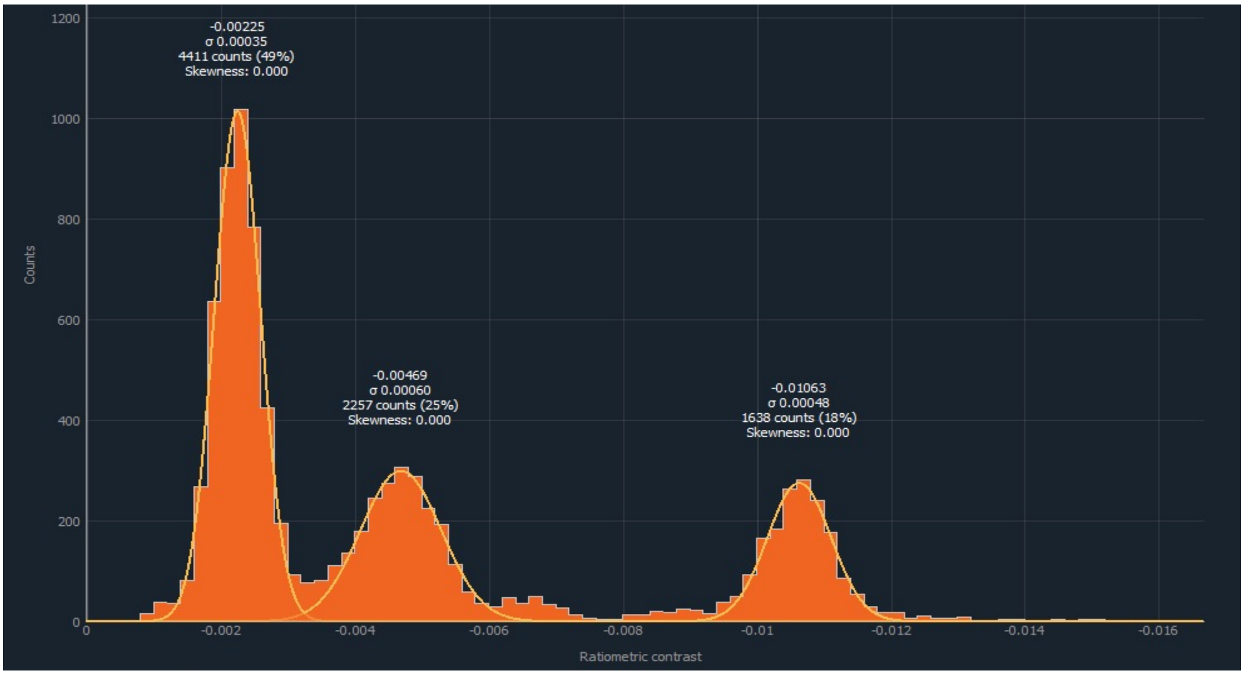

b

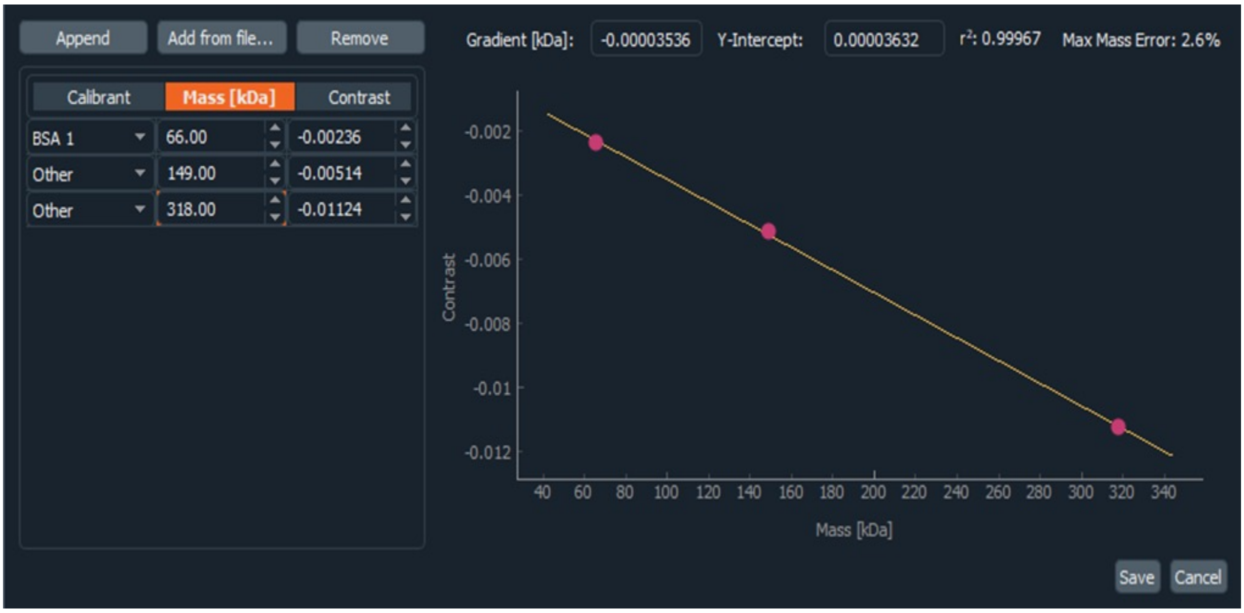

Supplementary Figure 10. Mass Photometer calibration. a Example of MP mass histogram integration of a mix BSA/Bevacizumab/GLDH. Peak have been integrated at their half-height. **b** Example of contrast-to-mass calibration using the mix BSA/Bevacizumab/GLDH.

a **RuvBL1ΔT127-E233**

MVHHHHHHLLVPRGSKIEEVKSTTKTQRIASHSHVKGLGLDESGLAKQAAS
GLVGQENAREACGVIVELIKSKKMAGRAVLLAGPPGTGKTALALAIAQELG
SKVPFCPMVGSEVYSTEIKKTEVLMENFRRAIGLRIKEGPPGIIQDVTLHDL
DVANARPQGGQDILSMMGQLMKPKKTEITDKLRGEINKVVNKYIDQGIA
ELVPGVLFVDEVHMLDIECFTYLHRALESSIAPIVIFASNRGNCVIRGTEDITS
PHGIPLDLLDRVMIIRTMLYTPQEMKQIIKIRAQTEGINISEEALNHLGEIGT
KTTLRYSVQLLTPANLLAKINGKDSIEKEHVEEISELFYDAKSSAKILADQQDK
YMK

b **SPAG1(622_926)_Flag**

MTFKALKEEGNQCVNDKNYKDALSKYSECLKINNKECAIYTNRALCYLKLC
QFEEAKQDCDQALQLADGNVKAFYRRALAHKGLKNYQKSLIDLNKVILLDP
SIIEAKMELEEVTRLLNLKDKTAPFNKEKERRKIEIQEVNEGKEEPGRPAGEV
SMGCLASEKGGKSSRSPEDPEKLPIAKPNNAYEFGQIINALSTRKDKEACAH
LLAITAPKDLPMFLSNKLEGDTFLLLIQSLKNNLIEKDPSLVYQHLLYLSKAER
FKMMLTLISKGQKELIEQLFEDLSDTPNNHFTLEDIQALKRQYELASDYKDD
DDK

c **RuvBL2ΔE134-E237**

MATVTATTKVPEIRDVTRIERIGAHSHIRGLGLDDALEPRQASQGMVGQLA
ARRAAGVVLEMIREGKIAGRAVLIAGQPGTGKTAIAMGMAQALGPDTPF
TAIAGSEIFSLEMSKTEALTQAFRRSIGVRIKEGPPGVVHTVSLHEIDVINSRT
QGFLALFSGDTGEIKSEVREQINAKVAEWREEGKAEIIPGVLFIDEVHMLDI
ESFSFLNRALESDMAPVLIMATNRGITRIRGTSYQSPHGIPIDLLDRLLIVSTT
PYSEKDTKQILRIRCEEEDVEMSEDAYTVLTRIGLETSLRYAIQLITAASLVCRK
RKGTEVQVDDIKRVYSLFLDESRSTQYMKEYQDAFLFNELKGETMDTSLEV
LFQ

d **PIH1D2(231_315)_STII**

MVKMPAYELKIVHDHSEKPLKIELKVELPGINSVSLCDLSVSEDDLLIEVSEK
YRLHLNLPKLIDTEMTTAKFIKEKSTLIITMPLVGSLEVLFQ

Supplementary Figure 11. Sequences of R2SP complex partners. a RuvBL1ΔT127-E233, **b** SPAG1(622_926)_Flag, **c** RuvBL2ΔE134-E237**, d** PIH1D2(231_315)_STII.

Supplementary Tables.

Supplementary Table 1. Protein-free droplet measurements with decreasing denaturant concentrations showing the impact of denaturant droplet concentration on Sharpness, Brightness and Signal parameters.

	Droplet concentration (M)	Sharpness (%)	Brightness (%)	Signal (%)
PBS	**2.9**	**5**	**73**	**0.03**
Urea	**5.4**	**3**	**44**	**0.05**
	**2.5**	**3**	**55**	**0.03**
	**1.75**	**5**	**63**	**0.03**
	**0.8**	**7**	**67**	**0.03**
Guanidine	**5.4**	**1**	**29**	**0.05**
	**2.5**	**3**	**47**	**0.04**
	**1.75**	**4**	**60**	**0.03**
	**0.8**	**4**	**62**	**0.03**
	**0.4**	**5**	**67**	**0.03**

Supplementary Table 2. Mass measurements and counts number after nMP and dMP measurements of BSA.

		**PBS**	**Urea 5,4M**		**Guanidine HCl 6M**	
			2h	16h	2h	16h
μ of gaussian fit (kDa)	Monomer (66.4 kDa)	75.0 ± 2.6	73.3 ± 1.5	71.7 ± 1.2	72.7 ± 2.1	73.7 ± 0.6
	Dimer (133 kDa)	144.3 ± 3.8	147.0 ± 4.4	142.3 ± 1.2	145.0 ± 3.5	142.3 ± 5.7
σ of gaussian fit (kDa)	Monomer (66.4 kDa)	8.2 ± 1.8	9.1 ± 0.9	7.4 ± 1.1	10.2 ± 1.4	11.7 ± 1.6
	Dimer (133 kDa)	8.4 ± 1.3	14.5 ± 1.4	8.1 ± 2.3	13.3 ± 0.8	13.5 ± 2.4

Supplementary Table 3. Identified cross-links in R2SP complex in the merged dataset (25, 100, 400 molar excesses combined). **a** Inter-protein cross-links **b** Intra-protein cross-links.

a

Inter-protein cross-links			
AbsPos1	Protein1	AbsPos2	Protein2
N-ter	RuvBL1	N-ter	RuvBL2
N-ter	RuvBL1	9	RuvBL2
16	RuvBL1	N-ter	RuvBL2
16	RuvBL1	9	RuvBL2
16	RuvBL1	26	RuvBL2
16	RuvBL1	67	RuvBL2
16	RuvBL1	132	RuvBL2
16	RuvBL1	179	RuvBL2
21	RuvBL1	N-ter	RuvBL2
21	RuvBL1	9	RuvBL2
21	RuvBL1	67	RuvBL2
21	RuvBL1	141	RuvBL2
21	RuvBL1	143	RuvBL2
21	RuvBL1	179	RuvBL2
25	RuvBL1	N-ter	RuvBL2
25	RuvBL1	67	RuvBL2
36	RuvBL1	N-ter	RuvBL2
36	RuvBL1	67	RuvBL2
47	RuvBL1	N-ter	RuvBL2
47	RuvBL1	317	RuvBL2
47	RuvBL1	319	RuvBL2
47	RuvBL1	327	RuvBL2
51	RuvBL1	317	RuvBL2
51	RuvBL1	327	RuvBL2
71	RuvBL1	N-ter	RuvBL2
71	RuvBL1	317	RuvBL2
179	RuvBL1	N-ter	RuvBL2
179	RuvBL1	179	RuvBL2
185	RuvBL1	N-ter	RuvBL2
185	RuvBL1	179	RuvBL2
192	RuvBL1	N-ter	RuvBL2
192	RuvBL1	9	RuvBL2
287	RuvBL1	N-ter	RuvBL2
333	RuvBL1	N-ter	RuvBL2
333	RuvBL1	9	RuvBL2
333	RuvBL1	67	RuvBL2
338	RuvBL1	N-ter	RuvBL2
356	RuvBL1	N-ter	RuvBL2
356	RuvBL1	9	RuvBL2
364	RuvBL1	327	RuvBL2
47	RuvBL1	202	SPAG1
287	RuvBL1	167	SPAG1
333	RuvBL1	135	SPAG1
333	RuvBL1	167	SPAG1
338	RuvBL1	167	SPAG1
N-ter	RuvBL2	20	SPAG1
N-ter	RuvBL2	85	SPAG1
N-ter	RuvBL2	121	SPAG1
N-ter	RuvBL2	123	SPAG1
317	RuvBL2	167	SPAG1
327	RuvBL2	7	SPAG1
327	RuvBL2	20	SPAG1
327	RuvBL2	121	SPAG1
327	RuvBL2	123	SPAG1
327	RuvBL2	200	SPAG1
327	RuvBL2	202	SPAG1

b

Intra-protein cross-links			
AbsPos1	Protein1	AbsPos2	Protein2
N-ter	RuvBL1	16	RuvBL1
N-ter	RuvBL1	185	RuvBL1
16	RuvBL1	21	RuvBL1
16	RuvBL1	25	RuvBL1
16	RuvBL1	36	RuvBL1
16	RuvBL1	179	RuvBL1
16	RuvBL1	185	RuvBL1
16	RuvBL1	192	RuvBL1
21	RuvBL1	36	RuvBL1
21	RuvBL1	179	RuvBL1
21	RuvBL1	185	RuvBL1
25	RuvBL1	36	RuvBL1
25	RuvBL1	179	RuvBL1
36	RuvBL1	21	RuvBL1
36	RuvBL1	287	RuvBL1
47	RuvBL1	71	RuvBL1
47	RuvBL1	287	RuvBL1
121	RuvBL1	179	RuvBL1
179	RuvBL1	185	RuvBL1
179	RuvBL1	192	RuvBL1
185	RuvBL1	185	RuvBL1
185	RuvBL1	192	RuvBL1
311	RuvBL1	338	RuvBL1
311	RuvBL1	356	RuvBL1
313	RuvBL1	354	RuvBL1
353	RuvBL1	364	RuvBL1
N-ter	RuvBL2	N-ter	RuvBL2
N-ter	RuvBL2	9	RuvBL2
N-ter	RuvBL2	26	RuvBL2
N-ter	RuvBL2	67	RuvBL2
N-ter	RuvBL2	132	RuvBL2
N-ter	RuvBL2	179	RuvBL2
N-ter	RuvBL2	265	RuvBL2
N-ter	RuvBL2	268	RuvBL2
N-ter	RuvBL2	317	RuvBL2
N-ter	RuvBL2	327	RuvBL2
8	RuvBL2	327	RuvBL2
9	RuvBL2	67	RuvBL2
9	RuvBL2	132	RuvBL2
9	RuvBL2	259	RuvBL2
9	RuvBL2	262	RuvBL2
9	RuvBL2	317	RuvBL2
9	RuvBL2	327	RuvBL2
67	RuvBL2	132	RuvBL2
132	RuvBL2	179	RuvBL2
4	SPAG1	35	SPAG1
7	SPAG1	35	SPAG1
17	SPAG1	49	SPAG1
20	SPAG1	31	SPAG1
20	SPAG1	49	SPAG1
49	SPAG1	72	SPAG1
49	SPAG1	82	SPAG1
49	SPAG1	129	SPAG1
85	SPAG1	121	SPAG1
85	SPAG1	124	SPAG1
85	SPAG1	129	SPAG1
89	SPAG1	121	SPAG1
89	SPAG1	123	SPAG1
89	SPAG1	135	SPAG1
121	SPAG1	129	SPAG1
121	SPAG1	135	SPAG1
123	SPAG1	135	SPAG1
123	SPAG1	202	SPAG1
124	SPAG1	135	SPAG1
129	SPAG1	135	SPAG1
164	SPAG1	202	SPAG1
167	SPAG1	202	SPAG1
177	SPAG1	202	SPAG1
202	SPAG1	244	SPAG1

Supplementary Table 4. Theoretical masses of studied complexes and their components. **a** Bovine serum albumin, **b** Alcool dehydrogenase, **c** L-glutamate dehydrogenase, **d** 20S human proteasome, **e** RuvBL1-RuvBL2-SPAG1-PIH1D2 complex.

a

Bovine serum albumin	
Monomer	66430 Da
Dimer	132860 Da
Trimer	199290 Da

b

Alcool dehydrogenase from Baker's yeast	
Monomer	36849 Da
Dimer	73698 Da
Trimer	110547 Da
Tetramer	147396 Da

c

L-glutamate dehydrogenase from bovine liver	
Monomer	55691 Da
Dimer	111382 Da
Trimer	167073 Da
Tetramer	222764 Da
Pentamer	278455 Da
Hexamer	334146 Da

d

20S human proteasome		
α	1	29556 Da
	2	25899 Da
	3	28433 Da
	4	29484 Da
	5	26411 Da
	6	27399 Da
	7	27887 Da
β	1	26489 Da
	2	22836 Da
	3	22949 Da
	4	29204 Da
	5	28480 Da
	6	25358 Da
	7	29965 Da
7-mer alpha		195069 Da
7-mer beta		185281 Da
28-mer		~ 700000 kDa

e

R2SP	
RuvBL1ΔT127-E233	40322 Da
RuvBL2ΔE134-E237	40743 Da
SPAG1(622_926)_Flag	36060 Da
PIH1D2(231_315)_STII	12144 Da
R1R2 hexamer	243195 Da
R1R2 dodecamer	486390 Da

Supplementary Table 5. Example of Eff_{XL} calculation on R2SP complex cross-linked with 400 molar excesses of DSBU.

Measurement replicate	Monomer Counts	Total counts	% of oligomeric states >1	Eff_{XL}
1	528	2006	$\frac{2006-528}{2006} * 100 = 74\ \%$	
2	892	5026	$\frac{5026-892}{5026} * 100 = 82\ \%$	**Mean = 77 % ± 5%**
3	690	2735	$\frac{2735-690}{2735} * 100 = 75\ \%$	

Supplementary Table 6. Example of SF_{XL} calculation on R2SP complex cross-linked with 400 molar excesses of DSBU.

	R2SP abundance (%)	SF_{XL}
**nMP** of R2SP	28	$\frac{0{,}17}{0{,}16} = \mathbf{0,6}$
**dMP** of 400:1 DSBU:R2SP	16	
